# Prognostic Impact of Copy Number Alterations’ Profile and AID/RAG Signatures in Acute Lymphoblastic Leukemia (ALL) with BCR::ABL and without Recurrent Genetic Aberrations (NEG ALL) Treated with Intensive Chemotherapy

**DOI:** 10.3390/cancers15225431

**Published:** 2023-11-15

**Authors:** Marta Libura, Karolina Karabin, Paweł Tyrna, Anna Czyż, Hanna Makuch-Łasica, Bożena Jaźwiec, Monika Paluszewska, Beata Piątkowska-Jakubas, Magdalena Zawada, Michał Gniot, Joanna Trubicka, Magdalena Szymańska, Katarzyna Borg, Marta Więsik, Sylwia Czekalska, Izabela Florek, Maria Król, Małgorzata Paszkowska-Kowalewska, Lidia Gil, Katarzyna Kapelko-Słowik, Elżbieta Patkowska, Agnieszka Tomaszewska, Krzysztof Mądry, Rafał Machowicz, Tomasz Czerw, Agnieszka Piekarska, Magdalena Dutka, Anna Kopińska, Grzegorz Helbig, Tomasz Gromek, Krzysztof Lewandowski, Marta Zacharczuk, Anna Pastwińska, Tomasz Wróbel, Olga Haus, Grzegorz Basak, Jerzy Hołowiecki, Przemysław Juszczyński, Ewa Lech-Marańda, Sebastian Giebel, Wiesław Wiktor Jędrzejczak

**Affiliations:** 1 Department of Hematology, Transplantation and Internal Medicine, Medical University of Warsaw, 02-091 Warsaw, Poland; karolina.karabin@o2.pl (K.K.); ptyrna@op.pl (P.T.); monika.paluszewska@uckwum.pl (M.P.); magdalena.szymanska3@uckwum.pl (M.S.); marta.wiesik@spartanska.pl (M.W.); mariakrol@spcsk.pl (M.K.); malgorzata.kowalewska@uckwum.pl (M.P.-K.); agnieszka.tomaszewska@wum.edu.pl (A.T.); krzysztof.madry@wum.edu.pl (K.M.); grzegorz.basak@wum.edu.pl (G.B.); wieslaw.jedrzejczak@wum.edu.pl (W.W.J.); 2 Department of Hematology, Blood Neoplasms and Bone Marrow Transplantation, Medical University of Wrocław, 50-137 Wrocław, Poland; aczyz@onet.eu (A.C.); bozena.jazwiec@umed.wroc.pl (B.J.); kks9999@wp.pl (K.K.-S.); martais91@onet.eu (M.Z.); tomasz_wrobel@wp.pl (T.W.); 3 Institute of Hematology and Transfusion Medicine, 02-776 Warsaw, Poland; hmakuch@ihit.waw.pl (H.M.-Ł.); kborg@ihit.waw.pl (K.B.); epatkowska@ihit.waw.pl (E.P.); pjuszczynski@ihit.waw.pl (P.J.); ewamaranda@wp.pl (E.L.-M.); 4 Department of Hematology, Jagiellonian University Medical College, 31-008 Cracow, Poland; bjakubas@wp.pl (B.P.-J.); mzawada@su.krakow.pl (M.Z.); sczekalska@su.krakow.pl (S.C.); iflorek@su.krakow.pl (I.F.); 5 Department of Hematology and Bone Marrow Transplantation, Poznań University of Medical Sciences, 61-701 Poznań, Poland; mgniot@ump.edu.pl (M.G.); lidia.gil@skpp.edu.pl (L.G.); krzysztof.lewandowski@skpp.edu.pl (K.L.); 6 Children’s Memorial Health Institute, 04-736 Warsaw, Poland; j.trubicka@ipczd.pl; 7 Maria Skłodowska-Curie National Research Institute of Oncology, Gliwice Branch, 44-102 Gliwice, Poland; tomasz.czerw@io.gliwice.pl (T.C.); jholowiecki@io.gliwice.pl (J.H.); sgiebel@io.gliwice.pl (S.G.); 8 Department of Hematology and Transplantology, Medical University of Gdańsk, 80-214 Gdańsk, Poland; agnieszka.piekarska@gumed.edu.pl (A.P.); magdalena.dutka@gumed.edu.pl (M.D.); 9 Department of Hematology and Bone Marrow Transplantation, Medical University of Silesia, 40-032 Katowice, Poland; akopinska@sum.edu.pl (A.K.); ghelbig@sum.edu.pl (G.H.); 10 Department of Hematooncology and Bone Marrow Transplantation, Medical University of Lublin, 20-081 Lublin, Poland; tgromek@spsk1.lublin.pl; 11 Department of Tumor Biology and Genetics, Medical University of Warsaw, 02-106 Warsaw, Poland; anna.pastwinska@wum.edu.pl; 12 Department of Clinical Genetics, Faculty of Medicine, Collegium Medicum in Bydgoszcz, Nicolaus Copernicus University, 87-100 Toruń, Poland; haus@cm.umk.pl

**Keywords:** acute lymphoblastic leukemia, primary aberrations, secondary aberrations, copy number alterations

## Abstract

**Simple Summary:**

Adult ALL is a highly aggressive blood cancer. Two classes of genetic aberrations are responsible for ALL: primary aberrations followed by secondary aberrations. Currently, primary aberrations are used for estimating patients’ risk in adult ALL. In this study, we reassessed the importance of primary and secondary copy number alterations (CNA) aberrations in intensively treated adult ALL patients in correlation to RAG/AID mutator enzyme expression. Primary aberrations alone specified the risk of 30% of patients. To define the prognosis of the remaining 70%, we identified high-risk and low-risk CNA profiles. We found the CNA profiles correlated with differential RAG/AID expression profiles. Furthermore, the outcome of CNA^neg^ adult ALL was stratified by AID expression. Thus, we suggested mechanisms linking secondary aberrations with patients’ outcomes and mutator enzymes. Finally, we propose a revised version of risk stratification in adult ALL patients which incorporates primary and secondary genetic lesions.

**Abstract:**

Adult acute lymphoblastic leukemia (ALL) is associated with poor outcomes. ALL is initiated by primary aberrations, but secondary genetic lesions are necessary for overt ALL. In this study, we reassessed the value of primary and secondary aberrations in intensively treated ALL patients in relation to mutator enzyme expression. RT-PCR, genomic PCR, and sequencing were applied to evaluate primary aberrations, while qPCR was used to measure the expression of RAG and AID mutator enzymes in 166 adult ALL patients. Secondary copy number alterations (CNA) were studied in 94 cases by MLPA assay. Primary aberrations alone stratified 30% of the patients (27% high-risk, 3% low-risk cases). The remaining 70% intermediate-risk patients included *BCR::ABL1*^pos^ subgroup and ALL lacking identified genetic markers (NEG ALL). We identified three CNA profiles: high-risk bad-CNA (CNA^high^/*IKZF1*^pos^), low-risk good-CNA (all other CNAs), and intermediate-risk CNA^neg^. Furthermore, based on RAG/AID expression, we report possible mechanisms underlying the CNA profiles associated with poor outcome: AID stratified outcome in CNA^neg^, which accompanied most likely a particular profile of single nucleotide variations, while RAG in CNA^pos^ increased the odds for CNA^high^/*IKZF1*^pos^ development. Finally, we integrated primary genetic aberrations with CNA to propose a revised risk stratification code, which allowed us to stratify 75% of *BCR::ABL1*^pos^ and NEG patients.

## 1. Introduction

B-cell acute lymphoblastic leukemia (B-ALL) is a biologically heterogeneous disease caused by specific genetic lesions leading to aberrant differentiation and proliferation of lymphoid progenitor cells [1]. Adult B-ALL is characterized by poor outcomes and a high rate of mortality when compared to children [2,3,4,5]. This is due to a particular genetic background and a higher rate of age-related comorbidities [6,7,8,9,10]. Although new therapies, as well as risk-adapted therapy protocols, led to a significant improvement in the outcome of adult ALL patients [11], the main reason for therapy failure in most cases is the emergence of new leukemic subclones leading to a relapse of the disease [12,13,14]. Thus, besides the standard risk group definition, the determination of the potential for leukemia’s evolution at the initial stage of the disease could be a key prognostication marker for those patients who would need further stratification.

A known hallmark of B-ALL development is the sequential acquisition of new genetic aberrations as a result of genetic instability and clonal evolution [13,15]. Chromosomal rearrangements and fusion genes, identified as primary aberrations, are the initiating events and key drivers of leukemogenesis, however, in most cases, they are insufficient for leukemia development. Secondary genetic events targeting B cell development genes are therefore an essential requirement for overt ALL [16,17]. These mutations, like copy number alterations (CNA; particularly gene deletions) and single-nucleotide variant (SNV) aberrations, have been frequently found within genes such as *IKZF1*, *CDKN2A/B*, and *PAX5* and have been reported to cooperate with each other and with primary lesions, leading to a more aggressive phenotype. Importantly, except for rare genetic subtypes, e.g., *MLL* rearrangements, the progression of high-risk B-ALLs depends mostly on mutator mechanisms introducing new CNA/SNV-type aberrations [14,18]. Not surprisingly, disease relapse is associated with both clonal diversification and a higher level of CNA mutation burden [19,20,21].

Recently, two lymphocyte-specific mutator enzymes, *RAG1/2* (recombination-activating gene) and *AID* (activation-induced cytidine deaminase), have been extensively studied in relation to genetic instability and oncogenicity in ALL. *RAG1* and *RAG2* genes encode recombinases, which introduce double-strand breaks into DNA during immunoglobulin/T-cell receptor genes’ rearrangement. However, aberrant targeting of *RAG1/2* to non-*IGH* sites contributes to the development of driver mutations and the clonal evolution of ALL [22]. The *AID* gene encodes cytidine deaminase, which generates point mutations in immunoglobulin genes in a process known as somatic hypermutations in germinal B-cells. *AID* was documented to be a driver of oncogenic mutations in lymphomas [23,24]. However, recent studies have shown that aberrant activation of *AID* by infectious signals may also accelerate mutagenic processes leading to childhood ALL [13,25,26,27]. The above-presented mutational mechanisms have been well described in pediatric ALL, but the data are insufficient for adult ALL. Although recent studies suggest the involvement of *RAG2*-mediated aberrant recombinations in the evolution of adult B-ALL: *t*(9;22)/*BCR::ABL1*^pos^ and *BCR::ABL*-like [28,29,30,31]; however, no comprehensive analyses on the expression of both enzymes across different genetic subgroups, in correlation to CNA status and clinical outcome, have been carried out in adult ALL so far.

Previous studies documented that both the mutation burden corresponding to clonal heterogeneity, and the type of the mutated gene (*IKZF1, CDKN2A/B*, or *PAX5)* may provide prognostic information [3,4,5,32,33,34]. However, controversial results have also been reported, particularly in adult ALL [2,35,36]. Although the factors that contribute to these discrepancies are not fully understood, they may be related to the complex nature of interactions between coexisting primary and/or secondary aberrations, different inclusion criteria (e.g., age, genetic background), or treatment protocols between independent trials. Because of these ambiguities, the fifth edition of WHO, as well as the ICC classification did not include a broad range of CNAs, as a diagnostic criterion for disease entities [37,38]. Importantly, most of these controversies apply to adult ALL. Whereas in pediatric ALL, the clinical significance of CNA has been well recognized and incorporated, e.g., into the integrated risk scoring system widely used in clinical trials, with the particular role of *IKZF1* deletions coexisting with other concomitant CNAs. This CNA profile was first described by Stanulla et al. as *IKZF*^plus^ and indicates a subgroup of patients with particularly poor outcomes (in our study, referred to as CNA^high^/*IKZF1*^pos^) [5,39,40]. As childhood ALL presents a cure rate of 85–90%, while most adult ALLs are poor-risk leukemias, it is important to understand better the possible role of mutational processes behind high-risk leukemias in adults.

Therefore, we carried out a detailed characterization of CNA profiles: CNA mutation burden, the type of mutated genes, and association with mutator enzyme *AID/RAG1/2* expression—to establish their correlation with prognosis in an adult B-ALL population treated with a standard intensive protocol according to PALG. Based on the obtained results, we proposed a combined revision of the genetic risk classification integrating CNA data with well-established primary aberrations, which enabled further prognostic stratification of *BCR::ABL1*^pos^ and NEG ALL patients. Parallelly, a comprehensive evaluation of *RAG1/2* and *AID* expression signatures in correlation with CNA profiles allowed for further characterization of mutational processes behind selected ALL subtypes associated with poor prognosis.

## 2. Materials and Methods

### 2.1. Patients’ Characteristics, Treatment, Material Collection, and Detection of Fusion Genes and Mutations by Molecular Analysis

A total of 166 patients with B-ALL were enrolled in the present study. The patients were diagnosed, and 161 patients met the criteria to be treated according to the Polish Adult Leukemia Group (PALG)-ALL5 or PALG-ALL6 protocols between 2007 and 2017 in six Polish hematology centers. The remaining 5 patients were disqualified from intensive therapy and received palliative treatment; their outcomes were not analyzed in this study. Allogenic hematopoietic stem cell transplantation (alloHSCT) from either an HLA-matched sibling or an unrelated donor was performed in 77/161 (47%) patients out of the whole group. 

The entire population was analyzed for common fusion genes: *BCR::ABL1*, *TEL::AML1*, *E2A::PBX1*, and *MLL::AF4,* using the RT-PCR protocol and primers designed according to the consensus of the European BIOMED-1 Concerted Action [41]. Subsequently, patients negative for the fusion genes listed above (102 patients) were screened for markers or surrogates of the *BCR::ABL1*-like phenotype (see details in Supplementary Materials). In parallel, cytogenetic analysis was performed on diagnostic bone marrow samples by PALG laboratories as previously described [42]. 

### 2.2. Treatment Protocols

The PALG ALL6 protocol encompassed all patients with newly diagnosed ALL. Patients were categorized for one of the first-line treatment options based on the subtype of the disease (ALL with or without the Philadelphia chromosome/*BCR::ABL1* rearrangement) and their age (either up to 55 years old or older). Younger patients adhered to a “pediatric” approach consisting of a pre-treatment phase, intensive induction, and consolidation therapy, followed by extended maintenance. Additionally, the protocols incorporated intensive and prolonged central nervous system prophylaxis. AlloHSCT was considered for patients with *BCR::ABL1*-positive disease and those with post-remission positive minimal residual disease (MRD) assessed by the multiparameter flow cytometry (MFC) method (MRD ≥ 0.1% after induction and/or >0.01% after consolidation). Compared to PALG ALL6, PALG ALL5 was a similar MRD-driven regimen, except for a less individualized approach to the treatment of older patients over 55 years old. Further information on the treatment protocols is provided in Supplementary Materials.

### 2.3. Gene Expression Analysis of Mutator Enzymes by Quantitative Real-Time PCR

Gene expression of *RAG1*, *RAG2,* and *AID* mutator enzymes was determined in the entire B-ALL cohort by real-time polymerase chain reaction (qPCR; see details in the Supplementary Methods). The whole group was segregated into 2 subgroups based on *RAG1*, *RAG2,* and *AID* expression levels at diagnosis, i.e., higher or lower than the median. As in our setting, there was a highly positive correlation between *RAG1* and *RAG2* enzymes, we used only the *RAG2* mRNA expression level for further analyses (Spearman’s rank correlation coefficient rho = 0.6; *p* < 0.05) [43].

### 2.4. CNA Detection: Multiplex Ligation-Dependent Probe Amplification (MLPA) and RT-PCR 

CNAs were evaluated using a multiplex ligation-dependent probe amplification (MLPA) assay. The SALSA MLPA P335-B2 ALL-IKZF1 (MRC-Holland, Amsterdam, The Netherlands) was used according to the manufacturer’s protocol in a group of 94 cases whose genetic material was available. The deletions in the loci of the following 7 genes were scored as deleted or non-deleted: *IKZF1*, *CDKN2A/B*, *PAX5*, *RB1*, *EBF1*, *ETV6*, and *BTG1* (see details in Supplementary Materials). Additionally, a cohort of 163 patients was evaluated for expression of 2 isoforms of *IKZF1* mRNA (Δ3–6, Δ1–7; primers for RT-PCR protocol described by Iacobucci I. et al. [44].

### 2.5. Statistical Analysis

Complete remission (CR) rate and probability of overall survival (OS), relapse-free survival (RFS), and disease-free survival (DFS) were the study endpoints. The CR rate was defined according to previously published criteria [4]. DFS was defined as the time from the achievement of CR to hematological relapse, death, or the last follow-up. OS was defined as the time from diagnosis to death or the last follow-up. 

Statistical differences between groups were tested with non-parametric tests. The Mann-Whitney U test was used for comparing continuous variables, the chi-squared test or Fisher exact test for categorical variables, and Spearman’s correlation test for correlating continuous data. The patients’ survival rates (OS, RFS, DFS) were compared with the log-rank test. In multivariate analyses, the hazard ratio was computed from the Cox proportional hazards model for survival rates (OS, RFS, DFS) and from the general linear model for categorical variables (CR). All statistical analyses were performed using the Statistica 13.3 software (TIBCO Software Inc., Palo Alto, CA, USA).

## 3. Results

### 3.1. Patients’ Characteristics; Frequency and Clinical Correlates of Primary Chromosomal Abnormalities

In the present study, 166 adults with B-ALL were investigated for the presence of fusion genes using the diagnostic workup according to the BIOMED-1 protocol. Comparisons of molecular results with cytogenetic data allowed for the identification of genetic subgroups according to the established primary chromosomal aberrations. The median age of the studied population was 37.5 years (range 18–70 years). According to the international criteria, 55 patients were classified into the very high-risk group, 94 into the high-risk group, and 17 into the standard-risk group. The demographic and clinical characteristics of the patients’ cohort are summarized in Table 1.

**Figure 1:**
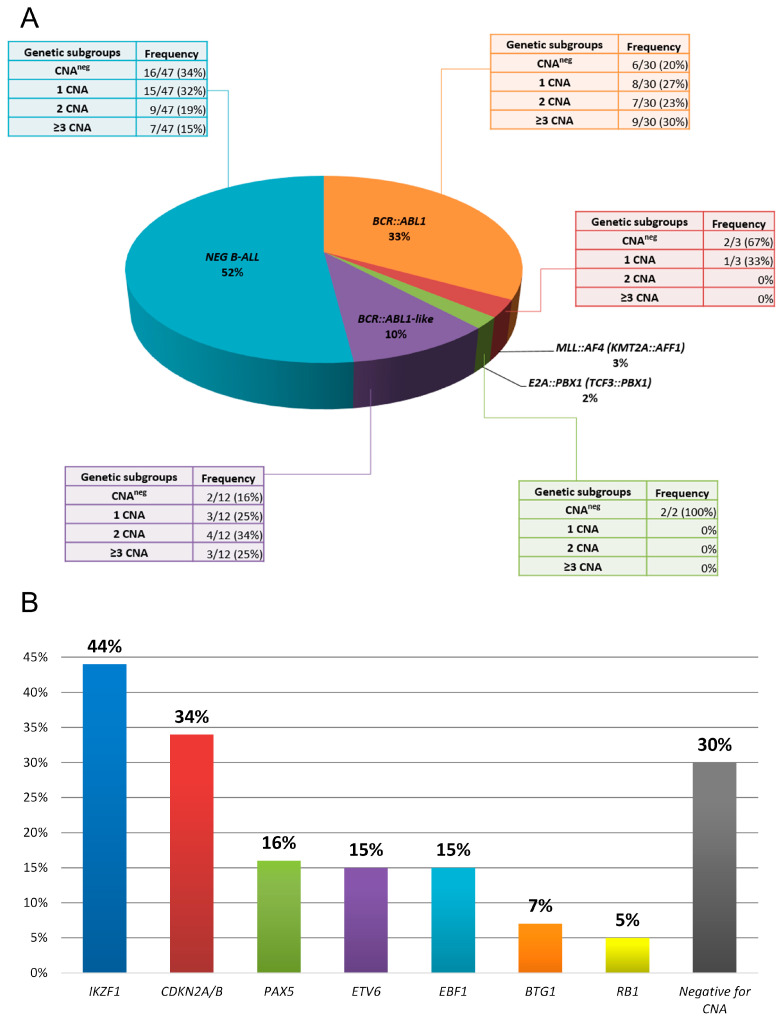
The frequency of primary and secondary aberrations in the patients’ cohort. (**A**) The frequency of primary aberrations in the patients’ cohort. The tables denote the frequencies of copy number alteration (CNA) mutation burden in the respective primary aberrations. (**B**) The frequency of genes with detected CNAs in the studied cohort. Some patients harbor more than one CNA, hence the sum exceeds 100%.

Out of the total 166 B-ALL patients analyzed, 55 (33%) had *BCR::ABL1*, 5 (3%) had *KMT2A::AFF1* (*MLL::AF4*), 4 (2%) had *TCF3::PBX1* (*E2A::PBX1*), and none had *ETV6::RUNX1* (*TEL::AML1*) fusion genes (Figure 1). One hundred-two patients (62%) were found negative for the common fusion genes analyzed according to the BIOMED-1 protocol. A subsequent analysis of these 102 patients identified 16 cases accompanied by the markers or surrogates of the *BCR::ABL1*-like phenotype (10% of the total population). All of the *BCR::ABL1*-like ALL represented *JAK::STAT2*–class aberrations: 8 patients with overexpression of *CRLF2* gene, 7 with *P2RY8::CRLF2* rearrangement, 1 with *CRLF2* gene point mutation, 6 with *JAK2* (see details in Table S1). 

When we matched the remaining group of 86 patients, who had no identified molecular aberrations, with cytogenetic data, 23 patients could be defined as a subgroup with poor risk genetic characteristics: 15 with complex karyotype, 6 hyperdiploid, 2 hypodiploid, and 1 with another aberration of poor risk. Out of the remaining 63 cases, 42 presented normal karyotypes, 1 had other aberrations of unknown significance, and 20 lacked the cytogenetic analysis. As this latter subgroup represented a “real world” population of ALL patients for whom the initial risk stratification is unknown, for the sake of further analyses in this manuscript, we grouped these patients under the label of “NEG ALL” (38%). 

### 3.2. Clinical and Biological Characteristics of RAG2 and AID Mutator Enzymes’ Expression—In Correlation with Primary Genetic Subgroups

In order to evaluate the potential for *RAG2-* and *AID*-mediated genetic instability, all patients were assigned into subgroups based on both enzymes’ mRNA expression levels that were higher or lower than the median (see Table 1B and Table S2). Thus, using the integrated *AID/RAG2* profile, we could identify 4 signatures of differential *AID* and *RAG2* expression (referred to here as “sig. 1–4): sig. 1 *AID*^low^/*RAG2*^low^ was found in 18.6% of ALL patients, who showed low expression levels of both enzymes; sig. 2 *AID*^high^/*RAG2*^low^ accompanied 32.7% of ALL population with high *AID* but low *RAG2*; sig. 3 *AID*^low^/*RAG2*^high^ was found in 31.4% of ALL, that showed high *RAG2* while little or no *AID*. Sig. 4 *AID*^high^/*RAG2*^high^ represented 17.3% of ALL who had both enzymes in abundance, suggesting co-synergic involvement of *AID* with *RAG2*. In summary, the highest frequency of sig. 3 (*AID*^low^/*RAG2*^high^) was found in the *BCR::ABL1*^pos^ subgroup, while the lowest one was in the NEG B-ALL (43% vs. 22%, *p* = 0.005). In contrast, a higher incidence of *AID*^high^/*RAG2*^low^ (sig. 2) was reported for the NEG B-ALL subgroup when compared to the *BCR::ABL1*^pos^ population (47% vs. 21.6%, *p* = 0.005). Detailed data on *AID/RAG2* profiles and their distribution in correlation to primary genetic aberrations are presented in the Supplementary Results. 

### 3.3. Clinical and Biological Characteristics of Secondary CNA Aberrations—In Correlation with Primary Genetic Subgroups

In the next step, we analyzed the characteristics of CNAs: CNA mutation burden and gene types in correlation with demographic data and B-ALL primary genetic aberrations in our study cohort (see Table 1C and Table S3). In summary, 1 or more deletions were observed in 66 out of 94 patients (70%), while 28 patients (30%) had no CNAs. The highest prevalence of CNAs was observed in the *BCR::ABL1* and *BCR::ABL1*-like subgroups (80% and 83.3%), followed by an intermediate one in the NEG B-ALL (68.6%), and the lowest in *MLL* gene rearrangement (33%). The detailed data on secondary CNA characteristics and correlation with genetic subgroups are further presented in the Supplementary Results.

### 3.4. Correlation of Secondary CNA Aberrations with RAG2/AID Signatures

Furthermore, in order to establish a functional link, we correlated the integrated *AID/RAG2* expression signatures with CNAs’ profiles (see Table 2 and Table S2). In summary, although we could confirm the presence of CNAs in each subgroup according to the integrated *AID/RAG2* signature profile, we report that the abundance of *RAG2* alone correlated rather with higher CNA levels and *IKZF1* deletions, particularly in the *BCR::ABL1*^pos^ context (*p* = 0.001), while a lower CNA mutation number or CNA^neg^ was more frequently accompanied by *AID*^high^/*RAG2*^low^, particularly in NEG ALL context, suggesting involvement of secondary mutations other than CNA, e.g., SNV (see Table S2 for details). Significantly, sig. 4, i.e., with parallel *AID* and *RAG2* abundance, was totally absent in CNA^neg^ patients, again emphasizing the link of both enzymes with CNA-type mutagenesis. Altogether, these results demonstrate a correlation of different CNA profiles with differential mutagenic properties linked to mutator enzymes or other mutagenic stimuli (*AID*^low^/*RAG2*^low^). The detailed description of correlation data between the integrated *AID/RAG2* expression signatures with CNAs’ profiles is further provided in the Supplementary Results.

### 3.5. The Outcome of Intensively Treated B-ALL Patients in Relation to Established Primary Aberrations

In the next part of the study, we verified the clinical outcomes of primary and secondary aberrations in the adult ALL population treated according to PALG protocol. Among the 166 patients analyzed for primary aberrations using molecular testing, 161 were eligible for intensive induction therapy, and 152 out of 161 were evaluated for response to induction, while 9 patients died before the remission evaluation. In the whole cohort, complete remission (CR) was achieved in 131/152 (86%) of the study population, which is consistent with the previous observations [45]. The median follow-up of the study group was 37.9 months and the median survival reached 20.5 months, which is comparable to results in other studies of similar populations. The estimated 4-year overall survival (OS) for the whole population was 34%, with a standard error of ±4%.

**Figure 2:**
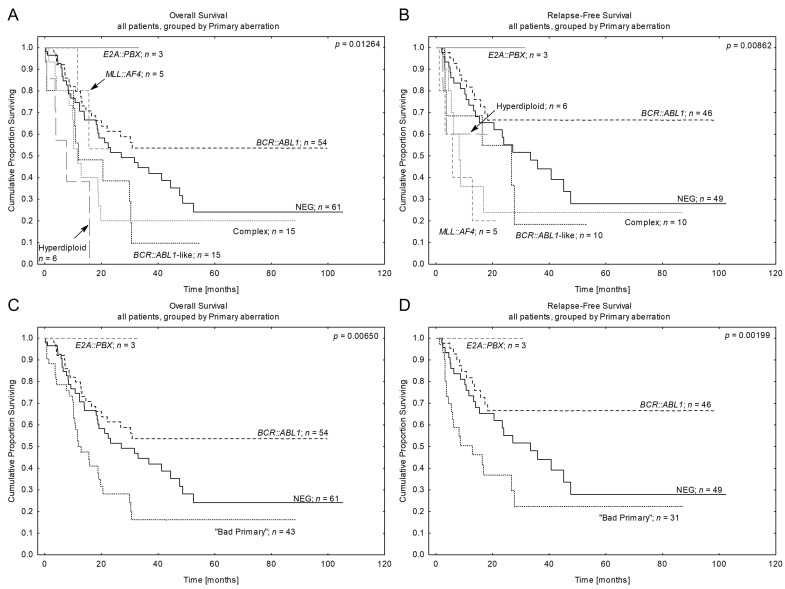
Patients’ outcomes according to the primary aberrations. (**A**,**B**) Kaplan-Meier estimates for the probability of overall survival and relapse-free survival in studied patients according to the primary aberration. (**C**,**D**) Kaplan-Meier estimates for the probability of overall survival and relapse-free survival in studied patients according to the revised code of the primary aberration. *n*—number of patients, *p*—*p*-value.

The impact of primary chromosomal aberrations on survival is shown in Table 3 and Figure 2A,B. Our data confirmed the poor 4-year outcome for patients harboring *MLL* rearrangements (OS 53 ± 25%), complex karyotype (OS 20 ± 10%), hyperdiploid karyotype (OS 0%), and in the *BCR::ABL1*-like subgroup (OS 10 ± 9%). As the last subgroup represented *JAK::STAT* pathway abnormalities, the poor prognosis of these patients has already been described in other cohorts [46]. All the cases with *E2A::PBX* exhibited a good outcome. Interestingly, in our study cohort, a relatively good outcome was also documented for the *BCR::ABL1*^pos^ subgroup (54 ± 8%). The remaining patients, for whom neither molecular nor cytogenetic abnormalities with established prognostic importance were detected, thus indicating obscure prognosis, were referred to here as NEG ALL and showed an intermediate risk (OS 32 ± 8%; Table 3). 

In multivariate analysis, the *BCR::ABL1* fusion gene independently predicted a lower risk of death, and hyperdiploid karyotype was independently associated with an increased death rate (OS for *BCR::ABL1*: *p* = 0.034 with HR: 0.55, 95% CI, 0.32–0.96; for hyperdiploid: *p* = 0.006 with HR: 3.91, 95% CI, 1.49–10.31). *BCR::ABL1* fusion independently predicted a lower incidence of relapse, while *MLL::AF4* independently associated with a higher risk of relapse (RFS for *BCR::ABL1: p* = 0.005 with HR: 0.34, 95% CI, 0.16–0.72; for *MLL* rearrangements: *p* = 0.013 with HR: 4.40, 95% CI, 1.37–14.13) after adjustment for WBC, age, and other primary aberrations in the total ALL series (Table 3B). 

Subsequently, based on the obtained data, we have assigned B-ALL patients to four categories: (1) “bad” primary aberrations, which grouped patients with all markers of poor prognosis (i.e., *MLL* rearrangements, *BCR::ABL*-like aberrations, complex, hyperdiploid, and hypodiploid karyotype), (2) “good” ones (*E2A::PBX*^pos^), as well as “intermediate” ones (IM), which included 2 subgroups: (3) *BCR::ABL1*^pos^ and (4) NEG ALL (Figure 2C,D; Table 3A). As the prognoses of the first and second categories of aberrations are well established (either “bad” or “good”), the prognoses of the 2 remaining IM subgroups: *BCR::ABL1*^pos^ and NEG ALL needed further clarification. Thus, we asked the question of whether CNA markers may help to identify cases with a high and low risk of disease progression. As *BCR::ABL1*^pos^ and NEG ALL genetic subgroups may constitute up to 70% of adult ALL, it was important for us to find relatively easily accessible prognostic markers like CNA for more relevant therapeutic decisions. In parallel, we evaluated the prognostic impact of CNA on the total ALL population as a historical group to compare with the results obtained by the others.

### 3.6. Prognostic Relevance of Secondary CNA Mutations in BCR::ABL1^pos^ and NEG ALL 

**Figure 3:**
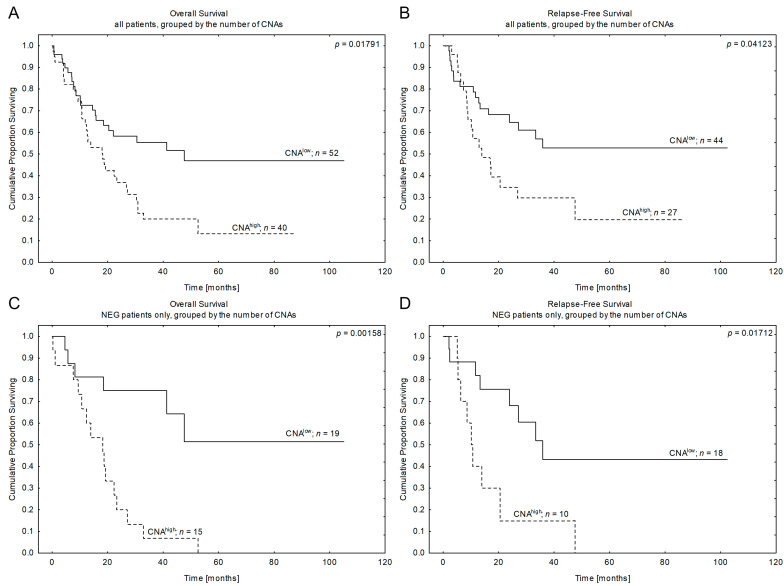
Patients’ outcomes according to the copy number alteration mutation burden. Kaplan-Meier estimates for the probability of overall survival and relapse-free survival in studied patients according to the number of copy number alterations (CNAs). CNA^low^—0–1 detected CNAs, CNA^high^—≥2 detected CNAs. (**A**,**B**)—Overall survival and relapse-free survival in all studied patients. (**C**,**D**)—Overall survival and relapse-free survival in NEG patients only. *n*—number of patients, *p*—*p*-value.

When we compared survival between CNA^pos^ and CNA^neg^ patients, we did not observe any significant differences (Table 4A). As the CNA^pos^ subgroup was heterogeneous with respect to the mutation burden and the gene type, we stratified patients according to the number of CNAs and compared their survival. After several intermediate analyses, which are presented in detail in the Supplementary Results section, we found rationale to stratify patients according to genetic instability level: the first subgroup was labeled as “CNA^low^” and included both CNA^neg^ and 1CNA patients, while the second subgroup was described as “CNA^high^” and included patients who harbored ≥2CNAs (see Table 1C for details). Comparing these two groups, we observed significant differences in survival in the NEG cohort as well as in the whole series: patients with CNA^high^ had lower OS and RFS rates than CNA^low^ patients (see Figure 3, Table 4A and Supplementary Results for details). Only a trend towards significance was observed in the *BCR::ABL1*^pos^ subgroup. The data suggested that a high mutation burden together with the cooperation of concomitant gene mutations may play a predominant role in prognosis rather than a single CNA.

**Figure 4:**
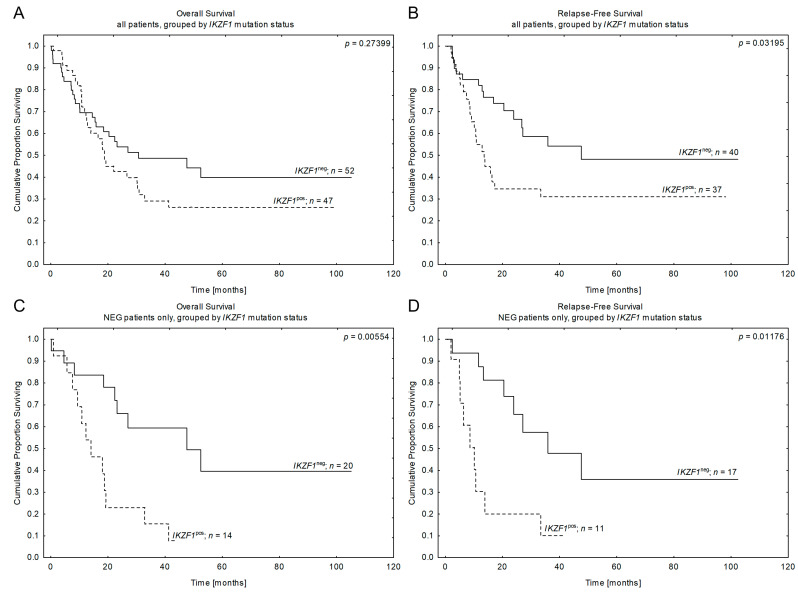
Patients’ outcome according to *IKZF1* mutation presence. Kaplan-Meier estimates for the probability of overall survival and relapse-free survival in studied patients according to *IKZF1* mutation status. (**A**,**B**)—Overall survival and relapse-free survival in all studied patients. (**C**,**D**)—Overall survival and relapse-free survival in NEG patients only. *n*—number of patients, *p*—*p*-value.

Thus, in the next step, we wanted to verify if any of the particular genes had a higher impact on survival than the others. We started with *IKZF1*, as it was the most frequent deletion in our study cohort, and found that its presence significantly correlated with the inferior outcomes of patients from NEG B-ALL. A trend toward statistical significance was observed for the *BCR::ABL1*^pos^ subgroup (see Figure 4 and Table 4A). For a detailed description of the obtained data, see Supplementary Results. In contrast, gene deletions like *CDKN2A/B* and *PAX5* had no prognostic impact in subgroup analysis or the entire series. 

Since the total *IKZF1*^pos^ population remained heterogeneous regarding the CNA burden, we assessed the difference between *IKZF1* deletion alone and *IKZF1* mutation coexisting with other CNAs (1CNA/*IKZF1*^pos^ vs. CNA^high^/*IKZF1*^pos^). Of note, Stanulla et al. were the first to describe the *IKZF1*^pos^ profile that co-occurred with deletions in *CDNK2A/B, PAX5,* or *PAR1* in the absence of *ERG* deletion, referring to it as the *IKZF*^plus^ subset, which defined poor prognostic subgroups among pediatric B-ALL [39]. In turn, in our study, the CNA^high^/*IKZF1*^pos^ subgroup included all *IKZF1*^pos^ patients accompanied by other gene deletions. As expected, the CNA^high^/*IKZF1*^pos^ patients showed worse survival when compared to the 1CNA/*IKZF1*^pos^ series. This difference was statistically significant in the *BCR::ABL1*^pos^ cohort and the entire series, while only a trend towards significance was found in the NEG subpopulation, probably due to a small number of 1CNA/*IKZF1*^pos^ patients. This suggests that the negative effect of *IKZF1* deletions within the total *IKZF1*^pos^ population was found exclusively in patients with CNA^high^/*IKZF1*^pos^ profile (see Figure S1, Table S4 for further data). A detailed description of these results is provided in the Supplementary Results.

Then, we attempted to verify if *IKZF1* had an adverse impact on other genes in the CNA^high^ population. Interestingly, when we compared the outcome of CNA^high^/*IKZF1*^pos^ vs. CNA^high^/*IKZF1*^neg^ series, the presence of *IKZF1* deletions conferred lower survival. Despite a relatively small number of cases in the subgroup analysis, these differences were statistically significant for NEG ALL, *BCR::ABL1*^pos^ cohort, and whole ALL series. These data may suggest again that the negative outcomes observed in the total CNA^high^ population may be mostly attributed to patients with CNA^high^/*IKZF1*^pos^ (see Table S4 and Figure S2 for further results). A detailed description of these data is provided in the Supplementary Results section.

Furthermore, as some studies reported [47,48] on the negative prognostic impact of *CDKN2A/B* deletions when accompanied by other deletions, particularly in *BCR::ABL1*^pos^ context, we compared the impact of *IKZF1* deletions in the CNA^high^/*CDNK2A/B*^pos^ cohort. Interestingly, *CDNK2A/B*^pos^ patients accompanied by *IKZF1* deletion showed worse outcomes when compared to the subgroup without *IKZF1*. Despite the relatively small number of patients, this effect was particularly evident in the *BCR::ABL1*^pos^ series (*p* = 0.01), suggesting that the coexistence of a *CDNK2A/B* gene deletion alone may not affect the outcome of the CNA^high^ population, but rather through cooperation with *IKZF1* deletions (Figure S3). A detailed description of these data is presented in the Supplementary Results section. 

In summary, these data document that CNA^high^*/IKZF1*^pos^ status is the predominant CNA profile with a clear negative impact on the treatment outcome in our series. As the dominant negative Ik6 isoform of *IKZF1* deletion was found in other study cohorts to be associated with the worst outcome, we compared the Ik6 prognostic power within the CNA^high^/*IKZF1^pos^* population [4,33]. Despite a small group of patients, we found a negative impact of Ik6 on relapse rate among the poor-risk CNA^high^*/IKZF1*^pos^ subgroup. 

Finally, we noticed that the prognostic impact of CNA^high^/*IKZF1*^pos^ varied in different age groups; a statistically significant difference in outcomes was obtained for younger patients (<40 y.), while no difference was observed for the older population (Figure S6). This suggests age-related comorbidities confounding the power of genetic markers in elderly patients, which can contribute to discrepancies in the prognostic impact of CNAs between clinical trials.

**Figure 5:**
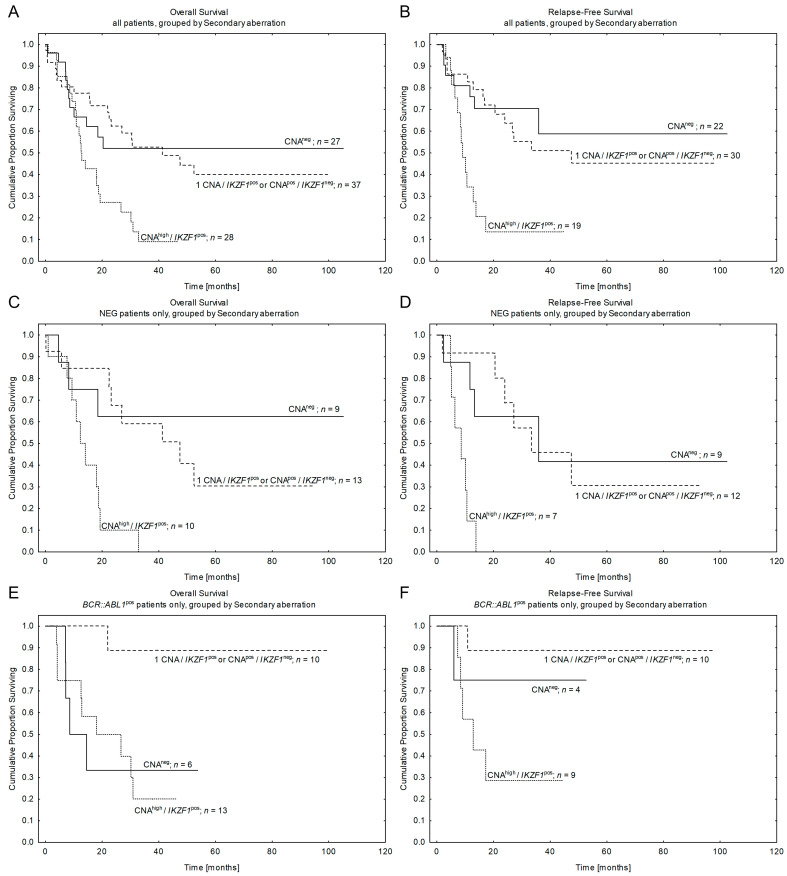
Patients’ outcomes according to the revised risk stratification by CNA profile. CNA^high^/*IKZF1*^pos^ represent bad-CNA profile, 1CNA/*IKZF1*^pos^ or CNA^high^/*IKZF1*^neg^ represent good-CNA profile. Kaplan-Meier estimates for the probability of overall survival and relapse-free survival in studied patients according to the revised code of secondary aberrations. (**A**,**B**)—all studied patients. OS: CNA^neg^ vs. good-CNA vs. bad-CNA *p* = 0.0566; good-CNA vs. bad-CNA *p* = 0.0026. RFS: CNA^neg^ vs. good-CNA vs. bad-CNA *p* = 0.0176; good-CNA vs. bad-CNA *p* = 0.0061. (**C**,**D**)—NEG patients only. OS: CNA^neg^ vs. good-CNA vs. bad-CNA *p* = 0.0212; good-CNA vs. bad-CNA *p* = 0.0055. RFS: CNA^neg^ vs. good-CNA vs. bad-CNA *p* = 0.0063; good-CNA vs. bad-CNA *p* = 0.0048. (**E**,**F**)—BCR::ABL1-positive patients only. OS: CNA^neg^ vs. good-CNA vs. bad-CNA *p* = 0.0152; good-CNA vs. bad-CNA *p* = 0.0032. RFS: CNA^neg^ vs. good-CNA vs. bad-CNA *p* = 0.0799; good-CNA vs. bad-CNA *p* = 0.0167. *n*—number of patients.

Thus, based on the above CNA data, we proposed a revised coding for risk stratification of adult B-ALL by CNA profile, which comprised 3 key subgroups, accompanied by different clinical impact: (1) “bad”-CNAs comprising CNA^high^/*IKZF1*^pos^ cases (OS 9 ± 6%; RFS 14 ± 9%); (2) relatively „good”-CNAs composed of 1CNA/*IKZF1*^pos^ or 1-4CNA/*IKZF1*^neg^ cases (OS 43 ± 9%; RFS 45 ± 9%); (3) the remaining CNA^neg^ ALL with unresolved prognosis–labeled as “intermediate”-CNAs (OS 43 ± 10%; RFS 59 ± 14%; *p* = 0.003 for bad-CNA vs. good-CNA; see Figure 5, Table 4A).

The revised CNA-risk index was valid for the whole series, but most importantly it stratified the prognosis of the two intermediate subgroups: 29% of NEG and 44% of *BCR::ABL1*^pos^ ALL were accompanied by bad-CNA, while 41% of NEG and 34% of *BCR::ABL1*^pos^ were associated with good-CNA profile (Figure 5C–F; Table 4A). Though the bad-CNA profile had equally poor prognosis both in the NEG and *BCR::ABL1*^pos^ subgroup, the good-CNA profile was associated with much better survival in the *BCR::ABL1*^pos^ patients (OS 89 ± 10%; RFS 89 ± 10%) when compared to the NEG ALL (*n* = 14, OS 28 ± 13%; RFS 31 ± 17%). It is possible that at least some good-CNA^pos^ cases from NEG ALL were in fact *BCR::ABL*-like patients, which are frequently accompanied by CNAs with poor prognosis by themselves.

Finally, the results of the multivariate Cox regression analysis were consistent with the results of the univariate analysis both in the NEG and *BCR::ABL* subgroups. CNA^high^ mutation burden, *IKZF1* deletion as well as the bad-CNA^high^/*IKZF1*^pos^ status were independent prognostic factors associated with worse OS and RFS in the NEG subgroup (OS *p* = 0.003 with HR: 4.95, 95% CI, 1.70–14.39 for CNA^high^; *p* = 0.011 with HR: 4.12, 95% CI, 1.37–12.33 for *IKZF1*^pos^; *p* = 0.007 with HR: 4.85, 95%CI, 1.56–15.09 for CNA^high^/*IKZF1*^pos^; Table 4B). In the *BCR::ABL1*^pos^ series, CNA^high^/*IKZF1*^pos^ had an independent prognostic impact on an increased risk of relapse (*p* = 0.030 with HR:7.65, 95% CI, 1.21–48.31), while good-CNAs, which lacked CNA^high^/*IKZF1*^pos^, independently predicted a lower risk of death (*p* = 0.04 with HR: 0.11, 95% CI, 0.01–0.90; Table 4B).

### 3.7. Combined Genetic Risk Classification by Revised Coding of Primary and Secondary Aberrations in Adult B-ALL

**Figure 6:**
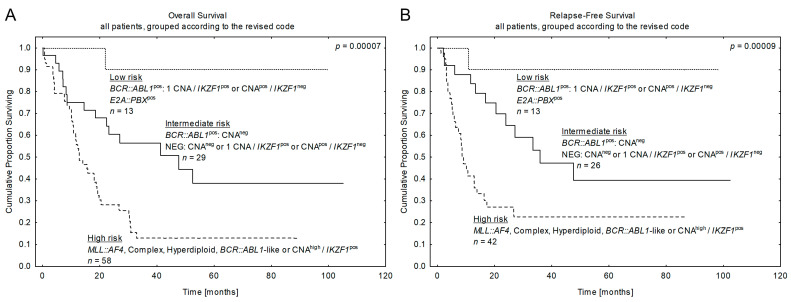
Patients’ outcomes according to the revised risk stratification by primary and secondary aberrations. Kaplan-Meier estimates for the probability of (**A**) overall survival and (**B**) relapse-free survival in studied patients according to the revised risk stratification code including the primary and the secondary aberration. *n*—number of patients, *p*—*p*-value.

Therefore, taking into account the data shown above, we could propose a novel combined risk-adapted classification for adult B-ALL: by incorporating the stratification of NEG and *BCR::ABL1*^pos^ subtypes by secondary CNA into the existing genetic classification based on primary aberrations with well-established prognostic effect (Figure 2C,D and Figure 5C–F) we could further refine prognosis of the entire adult ALL population. The entire cohort was divided into 3 subgroups, which were created by co-segregating different categories with comparable prognostic impact: intermediate-, low-, and high-risk features. The intermediate one (1) included intermediate-CNA^neg^ cases from both NEG and *BCR::ABL1*^pos^ subgroups as well as good-CNA cases from NEG ALL (1CNA/*IKZF1*^pos^ or 1-4CNA/*IKZF1*^pos^) identifying 33% adult ALL from our series; (2) the low-risk subgroup comprised *E2A::PBX*^pos^ cases and good-CNAs from *BCR::ABL1*^pos^ series (1CNA/*IKZF1*^pos^ and 1-4CNA/*IKZF1*^neg^) and contained 14% patients from our cohort; (3) while the 3rd one included both bad primary aberrations (*MLL::AF4*, *BCR::ABL*-like, complex, hyper/hypodiploid karyotype) and bad-CNAs (CNA^high^/*IKZF1*^pos^) from NEG or *BCR::ABL1*^pos^ ALL, and was associated with the worst outcome in 53% of total adult ALL (see Figure 6). 

These data indicate that more than half of adult ALL patients demonstrated poor outcomes, which has been revised compared to the frequency of 27% bad-risk ALL identified using only the well-established primary aberrations. The univariate and multivariate analyses for the combined revised risk stratification of adult ALL treated with PALG protocol are presented in Table 5.

**Figure 7:**
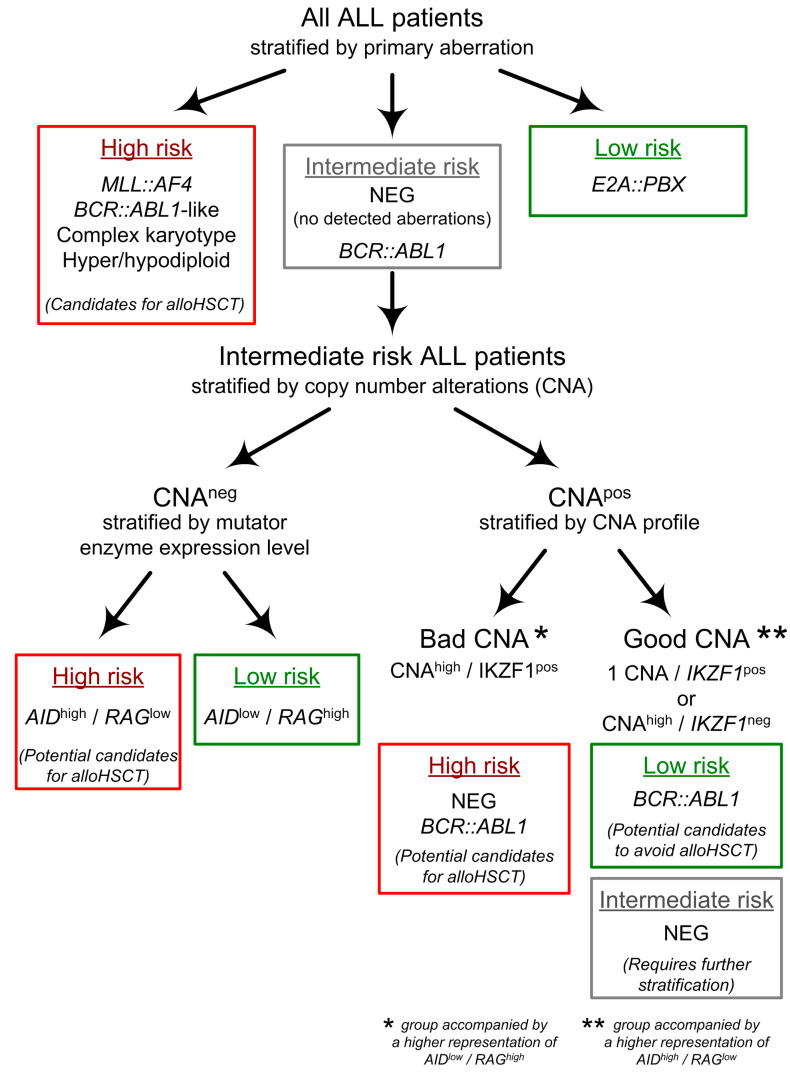
Schematic hierarchical definition of ALL genetic subgroups.

Finally, Figure 7 summarizes the flowchart of revised risk classification based on both primary and secondary aberrations, leading to a hierarchical definition of genetic subcategories. The identified CNA-based risk subgroups can be used to refine patient treatment according to a more detailed description of their leukemia.

### 3.8. Prognostic Relevance of RAG2 and AID Expression in CNA^neg^ and CNA^pos^ B-ALL Population

Interestingly, the three identified CNA profiles were associated not only with different outcomes but also with differential *AID/RAG2* signatures, suggesting a possible link between disease progression and the causal mutagenic mechanisms. Importantly, markers of CNA-based mutagenesis could not stratify CNA^neg^ cases, and these patients still constituted 31% and 20% of cases from the NEG and *BCR::ABL1*^pos^ subtypes, respectively. Thus, we tested whether the *AID/RAG2* signature was associated with disease progression in CNA^neg^ patients. Unfortunately, in this study, we did not have a chance to investigate SNVs or larger genomic lesions, which probably could drive the leukemogenesis of this subgroup. However, as SNV mutagenesis is related to *AID* activity, we speculated that a high *AID* expression may correlate with disease progression in CNA^neg^ cases. Indeed, despite a small number of cases, we report here a positive correlation between the *AID*^high^/*RAG2*^low^ expression signature and the incidence of adverse events in the CNA^neg^ subtype; patients who relapsed in 71% (5/7) were accompanied by high *AID* expression when compared to 20% (3/15) of those who remained in CR in the entire B-ALL series (*p* = 0.03; Table S5A). Such correlation was reversed for the CNA^pos^ series: only 20% (5/25) of *AID*^high^/*RAG2*^low^ signature was found among patients who eventually relapsed (*p* = 0.09). Interestingly, opposite data were reported for *RAG2* abundance in the CNA^neg^ series: a negative correlation with relapses (0/7) while a positive one with remission status (8/14; *p* = 0.01). 

These data prompted us to compare survival in the CNA^neg^ subgroup according to both *AID* and *RAG2* expression levels. Interestingly, high *AID* and low *RAG2* expressors were associated with low RFS and OS in this subgroup (Figure S4, Table S5B). In multivariate analysis, high *AID* and low *RAG2* independently predicted a higher incidence of relapse in the CNA^neg^ subset (Table S5C). These data, although based on a limited series of patients, allowed us to speculate that genomic markers other than CNA, which associate with *AID*^high^/*RAG2*^low^ signature may contribute to the outcome of CNA^neg^ patients. Thus, although we did not directly analyze the genetic background of CNA^neg^ ALL, we can assume that CNA^neg^ cases with *AID*^high^/*RAG2*^low^ signature, along with CNA^high^/*IKZF1*^pos^ cases may also represent a profile of “bad” secondary mutations. This again may help in the selection of new poor- and low-risk subgroups among CNA^neg^ patients, that can be used to refine patients’ treatment (about 17% of total ALL; see Figure 7). 

Finally, in the same manner, we decided to verify whether both enzymes correlate with clinical outcomes in the CNA^pos^ series, although an inverse pattern of correlation was assumed. This time, due to larger subgroups, the analysis could be performed separately for the NEG ALL and *BCR::ABL1*^pos^ series. Indeed, in the CNA^pos^ subgroup, *AID*^high^ was found to correlate with better survival in NEG ALL, while *AID*^low^/*RAG2*^high^ profile was associated with poor outcomes in *BCR::ABL1*^pos^ (Figure S5). Importantly, both *AID* and *RAG2* did not impact the outcome when evaluated independently from CNA status in the whole series, as well as in genetic subgroups.

## 4. Discussion

In this study, we reassessed the value of primary and CNA-type secondary aberrations in adult ALL treated with standard remission induction protocol according to PALG in relation to mutator enzyme *AID/RAG1/2* signatures. Our principal aim was to identify risk-defined subgroups and mutational processes underlying adult leukemias with poor outcomes. 

### 4.1. Revised Risk Index Based on Primary Aberrations Characterized the Prognosis of 30% of ALL Patients; Rationale for Further Stratification of NEG and BCR::ABL1^pos^ Subgroups

We have confirmed that established primary aberrations allowed 30% of adult ALL to be assigned to well-defined risk subgroups, either to the bad prognosis subgroup (28%; *KMT2A::AFF1* (*MLL::AF4*), *BCR::ABL1*-like phenotype, complex karyotype, and hyper-/hypodiploid karyotype); or the good prognosis subgroup with *TCF3::PBX1*; *E2A::PBX1* (2%). The remaining patients with *BCR::ABL1* or lacking identified genetic markers (NEG ALL) represented an intermediate outcome in need of further stratification. 

We proved a relatively good prognosis of *BCR::ABL1*^pos^ patients [49,50,51,52,53], however, we questioned whether all *BCR::ABL1*^pos^ patients may respond well to therapy. NEG ALL, which was negative for all investigated fusion genes and *BCR::ABL1*-like surrogate markers, was composed mainly of cases with normal karyotype (70%), or lacking cytogenetic analysis (30%). This subgroup thus represented a “real world” population of ALL patients with unknown prognoses. Importantly, although these two intermediate prognosis subgroups: NEG and *BCR::ABL1*^pos^ ALL covered 70% of adult ALL, their treatment outcome could not be further improved just by using conventional markers for initial risk stratification. Classic prognostic factors like age, WBC, fusion genes, and MRD are used for risk groups’ assignment [54]. However, routine practice shows that recurrent disease is not restricted only to the high-risk group. Additionally, the application of high-resolution techniques revealed that the genomic landscape particularly of NEG ALL is complex and heterogeneous [1,7,35,55]. Yet, the full spectrum of diagnostic workup according to the recent WHO/ICC classification is not always available, with many single aberrations lacking proven prognostic significance [37,38]. In relation to poor treatment outcomes of adult ALL, all these facts present a challenge for genetic classification based on risk. 

Therefore, we tested our ALL cohort for hidden genomic lesions: secondary copy number alterations (CNA), which cooperate with primary aberrations, and thus may impact the prognosis, and the choice of risk-adapted therapy. Their incidence reaches 60–70% across both childhood and adult ALL populations, representing an easily accessible marker for risk stratification [16,33]. However, as some results from different clinical trials were contradictory [2,3,4,36], we aimed to verify their clinical impact for intensively treated ALL patients according to PALG. As RAG and AID—known mutator enzymes—were documented to increase clonal heterogeneity [13,22], we decided to verify whether *RAG2/AID* expression signatures, may relate to CNA characteristics, their prognostic impact, and thus provide additional information on relapse potential.

Using the MLPA technique we detected CNAs in seven genes in 94 patients. Furthermore, *RAG2* and *AID* expression signatures were measured on a broader population of 166 patients with available RNA material. The distribution and frequencies of CNAs were generally consistent with those from other study cohorts in the whole series, as well as across genetic subgroups, with the highest CNA frequency in *BCR::ABL1*^pos^ (80% CNA) and *BCR::ABL1*-like subgroups (83% CNA), intermediate in NEG B-ALL (69% CNA), while the lowest in patients with *MLL* rearrangements [2,33]. This differential distribution already suggests a possible correlation with subtype-specific mutational processes and/or cooperation of concomitant gene aberrations.

### 4.2. RAG Is Associated with High CNA Mutation Burden, While AID Is Frequently Observed in CNA^low^ Patients

In order to elucidate a functional link between CNAs and the mutagenic process, we identified four different signatures of *AID* and *RAG2* expression and found their correlation with particular CNA profiles. Importantly, the signature with the highest expression of both enzymes was totally absent in the CNA^neg^ subgroup, re-emphasizing *AID* and *RAG2* involvement in CNA formation. The highest CNA mutation burden (≥3), *IKZF1* deletions, and particularly CNA^high^/*IKZF1*^pos^ profile were associated with high *RAG2* and low *AID* (*AID*^low^*/RAG2*^high^) signature. This suggests an increased RAG-mediated clonal diversity involved in the progression of these ALL subtypes. Interestingly, the highest incidence of the above CNAs as well as high *RAG2* expression was observed in *BCR::ABL1*^pos^ and *BCR::ABL*-like ALLs from our series. Increased *RAG*2 expression has been already described as a result of constitutive VDJ recombination activation by ABL and JAK-STAT kinase-activating lesions [30,31]. Additionally, our data indicate that *IKZF1* deletion by itself activates *RAG1/2* expression, thus increasing the risk for further deletions, which can lead to disastrous consequences [56].

In contrast to the above data, low CNA mutation burden (0–1), CNAs lacking *IKZF1*, and the absence of CNA (CNA^neg^) correlated with a higher representation of the *AID*^high^/*RAG2*^low^ signature, which signifies a possible role of AID-mediated mutagenesis underlying progression of these leukemias. AID is a member of the family of cytidine deaminases leading to substitution mutations, therefore its involvement in SNV-type rather than CNA-type mutagenesis may be expected. However, a co-synergic involvement of AID with RAG in CNA formation has been described as well [13]. Previous studies already reported higher *AID* expression in ALL lacking common primary aberrations (our NEG ALL subgroup) [57]. Here we expand these data by reporting prevalent *AID*^high^*/RAG*^low^ signature not only in NEG ALL but also in the CNA^neg^ series, suggesting its involvement in mutagenic processes other than CNA rearrangements in the absence of *RAG*. 

Interestingly, in contrast to *RAG2*, *AID* was found to be also upregulated by environmental factors, like repeated exposure to inflammatory stimuli, paralleling chronic infections in childhood [13,22,25,27]. Recent studies also describe a depleted microbiome as an accelerator of ALL development, while metabolites of a healthy microbiome prevented leukemia through AID inhibition [58,59,60]. Importantly, this interplay of AID with inflammation may be a factor stimulating clonal heterogeneity, e.g., in the infectious environment of immunodeficient ALL patients. Thus, while RAG has a well-established role in pro-B cells, expression of *AID* represents a recently discovered threat to lymphoid precursor genome integrity, but also a link to other cancers associated with chronic inflammation [13,26,61,62,63]. 

### 4.3. Prognostic Impact of CNAs Aberrations in Correlation with Data from Other Studies

Most importantly, CNA profiles in correlation with various *AID/RAG2* ratios—impacted treatment outcomes in our series. Thus, on the basis of the clinical data, we have proposed a revised CNA-based index stratifying prognosis of the intermediate subgroups from our series: NEG and *BCR::ABL* ALL. The CNA-based classification defined 3 subgroups with different CNA profiles and prognoses: bad-CNA (CNA^high^/*IKZF1*^pos^), relatively good-CNA (all other CNAs, lacking CNA^high^/*IKZF1*^pos^), and CNA^neg^ with an intermediate prognosis. 

We report here that bad-CNA: CNA^high^/*IKZF1*^pos^ is the most important CNA profile identifying patients with the worst outcome both in NEG and *BCR::ABL* subgroups of intensively treated adult ALL, and with the high *RAG2* expression as a causal driver of the mutagenic process. Additionally, despite a small group of patients, we report a particularly unfavorable outcome for the dominant negative Ik6 variant within the CNA^high^/*IKZF1*^pos^ subgroup. Our data are consistent with the observations of many authors [4,33]. Stanulla et al. were the first to describe the CNA^high^/*IKZF1*^pos^ profile as defining very poor prognosis in pediatric ALL (so-called *IKZF*^plus^ subgroup) [39]. Since then, these data have been confirmed in several studies both in childhood and adult ALL [3,5,33,40,64]. However, opposite results have also been published. Discrepancies mainly concerned adult ALL cohorts, while in childhood ALL the prognostic impact of CNA profiles was consistently well documented [2,35]. This points to age-related factors frequently confounding the prognostic power of genetic lesions. The prevalence of *IKZF1* deletions increases with age, thus studies omitting younger adults or adolescents will have a higher incidence of *IKZF1*^pos^ patients with an overlapping impact of age-related comorbidities [6,7]. Importantly, we confirmed this hypothesis in our study, showing that the prognostic impact of CNA^high^/*IKZF1*^pos^ was restricted to the younger population only (<40 y).

Another factor contributing to clinical discrepancies may relate to different group definitions based on genetic background. For example, many studies have chosen to analyze the impact of CNA^high^/*IKZF1*^pos^ among *BCR::ABL1*^neg^ patients [2,35]. In contrast, our NEG B-ALL population was not only negative for *BCR::ABL1* fusion but also excluded all other known primary aberrations, e.g., the majority of *BCR::ABL1*-like cases. 

### 4.4. Prognostic Impact of CNAs Aberrations in Particular Context of BCR::ABL1^pos^ Subgroup 

Importantly, the *BCR::ABL1*^pos^ series from our cohort was stratified not only by bad-CNA but also by good-CNA profile with favorite prognosis. Thus, the presence of good-CNAs could contribute to a relatively good outcome of our *BCR::ABL*1^pos^ series as a whole group. This observation is of importance, as some studies did not show any correlation between CNA and outcome for adult *BCR::ABL1*^pos^ ALL in the final multivariate analyses [2,33,64]. This may be due to the complex nature of chemotherapy resistance in *BCR::ABL1*^pos^ leukemias. According to the literature, most *BCR::ABL1*^pos^ patients relapse due to kinase domain mutations, which represent SNV-type aberrations, while 30–40% of patients relapse without kinase domain mutations [31]. We assume that the latter population may correspond to our bad-CNA with CNA^high^/*IKZF1*^pos^ profile, as *IKZF1* itself was documented to confer poor responsiveness to *ABL* kinase inhibitor therapy in the absence of mutation [65]. Interestingly, 5/7 patients who relapsed in our *BCR::ABL1*^pos^ series presented CNA^high^/*IKZF1*^pos^ deletions. Although we did not look for *ABL1* kinase domain point mutations in this subgroup, we assume that *RAG*-mediated CNA-type mutagenesis is more relevant in this subtype than SNV-type *ABL1* lesions, in line with high *RAG2* expression. However, overlap of CNA^high^/*IKZF1*^pos^ with SNV may also be considered, in line with observations of Koptyra et al. suggesting involvement of ROS-mediated SNV-type mutagenesis within ABL point mutations hotspot [66]. In contrast, good-CNA *BCR::ABL1*^pos^ patients from our series responded well to TKI treatment (see Figure 7).

Other studies report on *CDKN2A/B* deletions as a high-risk marker for adult *BCR::ABL*1^pos^ ALL [47,48]. Although on a limited series, we document, that *CDKN2A/B* deletions confer an adverse prognostic impact only when accompanied by *IKZF1* deletions. *CDKN2A/B* deletions were frequently accompanied by *IKZF1* deletions *in BCR::ABL1*^pos^ subgroups. Therefore, it is possible, that the poor prognosis of the CNA^high^/*CDKN2A/B*^pos^ subgroup could be attributed to the frequent coexistence of *IKZF1* with *CDKN2A/B* deletions as a part of CNA^high^/*IKZF1*^pos^ population.

In the NEG ALL subgroup, the clinical impact of good-CNA was labeled as “good”, although remained unresolved. This was mostly due to the unknown nature of other mutations, whose interplay with good-CNA could affect the prognostic power. 

### 4.5. Prognostic Impact of AID and RAG Expression in CNA^neg^ vs. CNA^pos^ Subgroups

Importantly, the evaluation of *AID* expression helped in the prognostic stratification of the CNA^neg^ subgroup (30% of total ALL). High *AID* expression has already been reported by Swaminathan et al. as a strong indicator of poor overall survival [13]. However, as part of this evaluation was performed on a pediatric group, here we present data for adult patients, specifying that the prognostic significance of *AID* was reserved only for CNA^neg^ patients. Although we do not know the mutation profile associated with the *AID*^high^*/RAG2*^low^ signature, our data signify distinct mutagenic processes accompanying disease progression in these high-risk CNA^neg^ patients, probably related to SNV-type lesions. Their association with inflammatory signals is also possible. 

In contrast, in the CNA^pos^ series high *RAG2* marked patients with poor outcomes, which resulted from the presence of CNA^high^/*IKZF1*^pos^ mutations. Thus, our data suggest that patients whose ALL involves increased *RAG2* or *AID*-mediated clonal heterogeneity exhibit a more aggressive disease because RAG and AID increase the probability of acquiring high-risk mutations, distinct in the context of CNA^pos^ and CNA^neg^ subgroups. 

### 4.6. Combined Revised Risk Classification of Adult ALL: CNA Data with Primary Aberrations Reclassify Prognostic Index of Adult ALL

Finally, on the basis of an extensive analysis of genetic data, we proposed a combined version of risk classification for intensively treated adult ALL, incorporating the CNA data into the risk index for primary aberrations. This is in contrast to the previous study by Moorman et al., who proposed a revised UKALL genetic risk classification based on key established primary aberrations as a stronger prognostic marker than secondary deletions [2]. Although the treatment protocol may affect prognostic factors, our study provides evidence that, at least for intensive treatment protocols such as PALG, besides *MLL* and hyperdiploid aberrations, secondary CNA aberrations stratify prognosis stronger than the established conventional factors. For example, these aberrations may confer a totally different outcome in patients with the same primary aberration. 

Unfortunately, we did not have a chance to correlate our results with MRD data for all patients. However, independently from the prognostic power of MRD by itself, one can imagine, that the blast cells possess the potential to evolve, which can change their genotype and phenotype. Thus, the negative MRD results assessed using techniques following initial profiles—may not always recapitulate the risk of relapse. Whereas initial CNA profiles inform on the risk of adverse events related to clonal evolution and heterogeneity. 

## 5. Conclusions

In summary, we present a pragmatic approach to select risk-defined ALL subgroups based on a simple categorization of CNA profiles. Integrating CNA data with the risk index by primary aberrations, allowed us to stratify the outcome of about 70% of adult ALL: into the poor-risk (53%), and favorable-risk populations (14%). This resulted in the reclassification of an additional 37% of patients who changed their status from IM-risk patients to favorable- or poor-risk, which may result in more relevant treatment stratification (see Figure 7). 

Importantly, specific CNA profiles correlated with different *AID/RAG2* signatures. We propose that these signatures contribute to the high-risk behavior of selected ALL subsets: *RAG* as an active driver increasing the odds for CNA^high^/*IKZF1*^pos^ development among CNA^pos^, while the abundance of *AID* indicates poor risk subset among CNA^neg^ ALL patients. This may be helpful to refine the treatment of CNA^neg^ patients. These data may also encourage the notion of risk-reducing antimutagenic interventions in the course of treatment (e.g., reinforcing anti-inflammatory background). 

Given the limitations of this study: its retrospective nature, the lack of a comprehensive SNV mutational profile at diagnosis, and relatively small subgroups, as well as scant data on mutational processes in adult ALL compared to the vast data in childhood leukemias—further investigations in this field are warranted.

## Figures and Tables

**Table 1:** Patients’ characteristics according to the primary aberrations, secondary aberrations and mutator enzyme expression. (**A**) Patients’ characteristics according to the primary aberration. (**B**) Patients’ characteristics according to RAG2 and AID expression. (**C**) Patients’ characteristics according to the number of CNAs and IKZF1 mutation status.

(A)													
	Total		NEG		*BCR::ABL1*	“Bad Primary”						*E2A::PBX*	
						*BCR::ABL1*-like	*MLL::AF4*		Complex	Hyperdiploid	All “Bad Primary” ^‡^		
	*n* = 166		*n* = 63 (38%)		*n* = 55 (33%)	*n* = 16 (10%)	*n* = 5 (3%)		*n* = 15 (9%)	*n* = 6 (4%)	*n* = 44 (27%)	*n* = 4 (2%)	
**Age**													
≤37 years (%)	83 (50%)		33 (52%)		24 (44%)	6 (38%)	5 (100%)		8 (53%)	3 (50%)	23 (52%)	3 (75%)	
>37 years (%)	83 (50%)		30 (48%)		31 (56%)	10 (62%)	0 (0%)		7 (47%)	3 (50%)	21 (48%)	1 (25%)	
**Gender**													
Female (%)	57 (34%)		15 (24%)		24 (44%)	7 (44%)	0 (0%)		5 (33%)	3 (50%)	16 (36%)	2 (50%)	
Male (%)	109 (66%)		48 (76%)		31 (56%)	9 (56%)	5 (100%)		10 (67%)	3 (50%)	28 (64%)	2 (50%)	
**Immunological subtype**													
prepreB (%)	22 (13%)		11 (17%)		3 (5%)	1 (6%)	3 (60%)		3 (21%)	1 (17%)	8 (19%)	0 (0%)	
preB (%)	42 (25%)		17 (27%)		13 (24%)	4 (25%)	2 (40%)		4 (29%)	1 (17%)	11 (26%)	1 (25%)	
common (%)	101 (62%)		35 (56%)		39 (71%)	11 (69%)	0 (0%)		7 (50%)	4 (67%)	24 (56%)	3 (75%)	
**Median of WBC, ×10^9^/L**	17.0		13.5		26.0	28.7	127.9 ^#^		9.2	4.6	10.9	24.3	
**Rate of CD20 positive, %**	18.0		14.0		28.0	31.0	0.5		35.0	25.0	25.0	9.5	
**Rate of CD52 positive, %**	18.0		18.0		22.0	43.0	8.7		12.0	5.5	14.7	62.0	
**Risk group according to PALG**													
very high risk (%)	55 (33%)		0 (0%)		55 (100%)	0 (0%)	0 (0%)		0 (0%)	0 (0%)	0 (0%)	0 (0%)	
high risk (%)	94 (57%)		54 (86%)		0 (0%)	16 (100%)	5 (100%)		13 (87%)	3 (50%)	38 (86%)	2 (50%)	
standard risk (%)	17 (10%)		9 (14%)		0 (0%)	0 (0%)	0 (0%)		2 (13%)	3 (50%)	6 (14%)	2 (50%)	
**MRD status after first induction**													
positive (%)	45 (38%)		13 (30%)		19 (53%)	5 (56%)	0 (0%)		6 (43%)	1 (20%)	13 (37%)	0 (0%)	
negative (%)	73 (62%)		31 (70%)		17 (47%)	4 (44%)	5 (100%)		8 (57%)	4 (80%)	22 (63%)	3 (100%)	
**Induction regimen**													
PALG5 (%)	15 (9%)		4 (6%)		6 (11%)	0 (0%)	0 (0%)		2 (13%)	1 (17%)	3 (7%)	2 (50%)	
PALG6 (%)	145 (88%)		58 (92%)		46 (84%)	15 (100%)	5 (100%)		13 (87%)	5 (83%)	40 (93%)	1 (25%)	
PALG modified (%)	5 (3%)		1 (2%)		3 (5%)	0 (0%)	0 (0%)		0 (0%)	0 (0%)	0 (0%)	1 (25%)	
**AlloHSCT in first complete response**													
yes (%)	77 (47%)		27 (44%)		29 (53%)	7 (44%)	5 (100%)		6 (40%)	1 (17%)	19 (43%)	2 (50%)	
no (%)	88 (53%)		35 (56%)		26 (47%)	9 (56%)	0 (0%)		9 (60%)	5 (83%)	25 (57%)	2 (50%)	
**(B)**													
	**Total**		***RAG2* Expression**						***AID* Expression**				
			**Low**			**High**	***p***		**Low**		**High**	***p***	
	***n* = 166**		***n* = 83**			***n* = 78**			***n* = 78**		***n* = 79**		
**Median age, years**	37.5		40.0			37.0	0.4817 *		35.5		41.0	0.2802 *	
**Gender**							0.5191 ^†^					0.1440 ^†^	
Female (%)	57		28 (34%)			27 (35%)			31 (40%)		24 (30%)		
Male (%)	109		55 (66%)			51 (65%)			47 (60%)		55 (70%)		
**Primary aberration**													
NEG	63		37 (62%)			23 (38%)	**0.0478** ^a^		22 (38%)		36 (62%)	**0.0242** ^a^	
*BCR::ABL1*	55		21 (39%)			33 (61%)	**0.0224** ^b^		30 (57%)		23 (43%)	0.2156 ^b^	
*BCR::ABL1-like*	16		8 (53%)			7 (47%)			7 (50%)		7 (50%)		
*MLL::AF4*	5		2 (40%)			3 (60%)			5 (100%)		0 (0%)		
Complex	15		8 (53%)			7 (47%)			7 (47%)		8 (53%)		
Hyperdiploid	8		5 (83%)			1 (17%)			3 (50%)		3 (50%)		
*E2A::PBX*	4		1 (25%)			3 (75%)			3 (75%)		1 (25%)		
**Immunological subtype**							0.4641 ^c^					**0.0420** ^c^	
prepreB (%)	22		13 (59%)			9 (41%)			14 (67%)		7 (33%)		
preB (%)	42		18 (46%)			21(54%)			14 (36%)		25 (64%)		
common (%)	101		51 (52%)			48 (48%)			50 (52%)		46 (48%)		
**Median of WBC, ×10^9^/L**	17.0		10.2			23.0	0.0559 *		21.0		12.9	0.0589 *	
**Rate of CD20 positive, %**	18.0		22.0			20.0	0.5278 *		11.0		30.0	0.3524 *	
**Rate of CD52 positive, %**	18.0		22.5			17.5	0.5950 *		22.5		17.0	0.1320 *	
**Risk group according to PALG**							0.0681 ^†^					0.1691 ^†^	
very high risk (%)	55		21 (39%)			33 (61%)			30 (57%)		23 (43%)		
high risk (%)	94		52 (57%)			39 (43%)			38 (43%)		50 (57%)		
standard risk (%)	17		10 (63%)			6 (37%)			10 (63%)		6 (37%)		
**MRD status after first induction**							0.4792 ^†^					0.1463 ^†^	
positive (%)	45		24 (55%)			20 (45%)			18 (43%)		24 (57%)		
negative (%)	73		36 (52%)			33 (48%)			38 (55%)		31 (45%)		
**Induction regimen**													
PALG5 (%)	15		8 (57%)			6 (43%)			4 (29%)		10 (71%)		
PALG6 (%)	145		73 (52%)			68 (48%)			71 (52%)		66 (48%)		
PALG modified (%)	5		1 (20%)			4 (80%)			3 (60%)		2 (40%)		
**AlloHSCT in first complete response**													
yes (%)	77		36 (49%)			38(51%)			34 (47%)		38 (53%)		
no (%)	88		47 (54%)			40 (46%)			44 (52%)		41 (48%)		
**(C)**													
	**Total**	**CNA Presence**			**CNA Mutation Burden**			***IKZF1* Mutation Status**			***IKZF1*^pos^ Patients Only ^d^**		
		**CNA^neg^**	**CNA^pos^**	***p***	**CNA^low^ (0–1 CNA)**	**CNA^high^ (≥2 CNAs)**	***p***	***IKZF* ^neg^**	***IKZF* ^pos e^**	***p***	**1 CNA**	**CNA^high^**	***p***
	***n* = 94**	***n* = 28** **(30%)**	***n* = 66** **(70%)**		***n* = 53** **(56%)**	***n* = 41** **(44%)**		***n* = 53** **(52%)**	***n* = 49** **(48%)**		***n* = 12** **(29%)**	***n* = 29** **(71%)**	
**Median age, years**	37.0	33.0	39.5	**0.0227** *	36.0	39.0	0.1128 *	35.0	41.0	**0.0152** *	41.0	45.0	0.6466 *
**Gender**				0.5383 ^†^			0.3658 ^†^			0.3789 ^†^			0.5365 ^†^
Female (%)	36	11 (31%)	25 (69%)		19 (53%)	17 (47%)		21 (58%)	17 (42%)		4 (27%)	11 (73%)	
Male (%)	58	17 (29%)	41 (71%)		34 (59%)	24 (41%)		32 (50%)	32 (50%)		8 (31%)	18 (69%)	
**Primary aberration**													
NEG	35 (37%)	11 (31%)	24 (69%)	0.7887 ^a^	20 (57%)	15 (43%)	0.9089 ^a^	21 (60%)	14 (40%)	0.2402 ^a^	4 (29%)	10 (71%)	0.9437 ^a^
*BCR::ABL1*	30 (32%)	6 (20%)	24 (80%)	0.1554 ^b^	14 (47%)	16 (53%)	0.1934 ^b^	10 (27%)	27 (73%)	**0.0001** ^b^	6 (30%)	14 (70%)	0.9200 ^b^
*BCR::ABL1-like*	12 (13%)	2 (17%)	10 (83%)		5 (42%)	7 (58%)		6 (50%)	6 (50%)		2 (33%)	4 (67%)	
*MLL::AF4*	3 (3%)	2 (67%)	1 (33%)		3 (100%)	0 (0%)		3 (100%)	0 (0%)		0 (0%)	0 (0%)	
Complex	6 (6%)	2 (33%)	4 (67%)		3 (50%)	3 (50%)		5 (71%)	2 (29%)		0 (0%)	1 (100%)	
Hyperdiploid	6 (6%)	3 (50%)	3 (50%)		6 (100%)	0 (0%)		6 (100%)	0 (0%)		0 (0%)	0 (0%)	
*E2A::PBX*	2 (2%)	2 (100%)	0 (0%)		2 (100%)	0 (0%)		2 (100%)	0 (0%)		0 (0%)	0 (0%)	
**Immunological subtype**				0.2555 ^c^			0.2765 ^c^			0.8579 ^c^			0.9534 ^c^
prepreB (%)	11	5 (45%)	6 (55%)		7 (64%)	4 (46%)		9 (82%)	2 (18%)		0 (0%)	2 (100%)	
preB (%)	26	10 (38%)	16 (62%)		17 (65%)	9 (35%)		16 (53%)	14 (47%)		3 (30%)	7 (70%)	
common (%)	57	13 (23%)	44 (77%)		29 (51%)	28 (49%)		28 (46%)	33 (54%)		9 (31%)	20 (69%)	
**Median of WBC, ×10^9^/L**	15.5	13.7	17.9	0.8178 *	13.6	23.6	0.0939 *	13.8	19.0	0.3394 *	7.2	24.4	0.1188 *
**Rate of CD20 positive, %**	18.0	11.0	18.0	0.7008 *	10.0	20.0	0.2409 *	18.0	20.0	0.9968 *	6.2	19.0	0.1939 *
**Rate of CD52 positive, %**	22.5	15.0	26.2	0.2507 *	26.0	21.5	0.4730 *	22.0	18.0	0.6487 *	82.0	18.0	0.0502 *
**Risk group acc. to PALG**				0.0567 ^†^			0.0561 ^†^			**0.0005** ^†^			0.7786 ^†^
very high risk (%)	30	6 (20%)	24 (80%)		14 (47%)	16 (53%)		10 (27%)	27 (73%)		6 (30%)	14 (70%)	
high risk (%)	54	16 (30%)	38 (70%)		30 (56%)	24 (44%)		35 (64%)	20 (36%)		5 (26%)	14 (74%)	
standard risk (%)	10	6 (60%)	4 (40%)		9 (90%)	1 (10%)		8 (80%)	2 (20%)		1 (50%)	1 (50%)	
**MRD after 1st induction**				**0.0486** ^†^			**0.0003** ^†^			**0.0019** ^†^			**0.0149** ^†^
positive (%)	25	5 (20%)	20 (80%)		9 (36%)	16 (64%)		9 (31%)	20 (69%)		2 (13%)	14 (87%)	
negative (%)	42	18 (43%)	24 (57%)		34 (81%)	8 (19%)		30 (68%)	14 (32%)		7 (58%)	5 (42%)	
**Induction regimen**													
PALG5 (%)	8	2 (25%)	6 (75%)		6 (75%)	2 (25%)		3 (38%)	5 (63%)		3 (60%)	2 (40%)	
PALG6 (%)	83	25 (30%)	58 (70%)		46 (55%)	37 (45%)		49 (54%)	42 (46%)		9 (26%)	25 (74%)	
PALG modified (%)	2	0 (0%)	2 (100%)		0 (0%)	2 (100%)		0 (0%)	2 (100%)		0 (0%)	2 (100%)	
**AlloHSCT in first CR**													
yes (%)	40	14 (35%)	26 (65%)		29 (73%)	11 (27%)		25 (56%)	20 (44%)		8 (53%)	7 (47%)	
no (%)	54	14 (26%)	40 (74%)		24 (44%)	30 (56%)		28 (49%)	29 (51%)		4 (15%)	22 (85%)	

^‡^ Two hypodiploid cases are included in the “bad genetics” group but are not included in the preceding subgroups. ^†^ computed by chi-squared or Fisher exact test. * computed by Mann-Whitney U test. ^#^ WBC in *MLL::AF4* significantly higher than in NEG, Complex, and Hyperdiploid patients (ANOVA *p* = 0.0038, Tukey HSD test *p* = 0.0163, *p* = 0.0470 and *p* = 0.0271, respectively. Other comparisons not significant). ^a^ computed by chi-squared test: NEG vs. all other cases. ^b^ computed by chi-squared test: *BCR::ABL1* vs. all other cases. ^c^ computed by chi-squared test: preB vs. all other cases. ^d^ “MLPA group”: this series includes *IKZF1*^pos^ cases identified using MLPA (37) and RT-PCR (2) in a group of 94 patients, for whom other gene deletions were analyzed using MLPA; this series was used for evaluation of patients’ characteristic (see also Table S2). ^e^ “MLPA+RT-PCR group”: this series includes *IKZF1*^pos^ cases identified using MPLA (37) and RT-PCR (10); This series was used for survival analyses. Abbreviations: *RAG2*, Recombination Activating Gene 2; *AID*, Activation Induced Cytidine Deaminase; WBC, White Blood Cell count; PALG, Polish Adult Leukemia Group; CNA, copy number alterations.

**Table 2:** Prevalence of markers of CNA-type genetic instability: mutational burden, gene type—in subgroups according to mutator enzyme expression.

Total B-ALL					
	*RAG2* Expression		*AID* Expression		*p* ^†^
	Low *n* = 43	High *n* = 48	Low *n* = 54	High *n* = 36	
**CNA**					
CNA^neg^	18 (67%)	9 (33%)	15 (54%)	13 (46%)	
CNA^pos^	25 (39%)	39 (61%)	36 (61%)	23 (39%)	*RAG2*: CNA^neg^ vs. CNA^pos^: 0.0160
1 CNA	9 (39%)	14 (61%)	11 (50%)	11 (50%)	
CNA^high^	16 (39%)	25 (61%)	25 (68%)	12 (32%)	*RAG2*: CNA^neg^ vs. CNA^high^: 0.0257
**IKZF1 deletion**					
*IKZF1* ^neg^	31 (60%)	21 (40%)	24 (46%)	28 (54%)	*RAG2*: 0.0109
*IKZF1* ^pos^	16 (34%)	31 (66%)	31 (72%)	12 (28%)	*AID*: 0.0108
***CDKN2A/B*** **deletion**					*RAG2*: 0.0013
*CDKN2A/B* ^neg^	33 (56%)	26 (44%)	36 (63%)	21 (37%)	
*CDKN2A/B* ^pos^	10 (31%)	22 (69%)	15 (50%)	15 (50%)	
***PAX5*** **deletion**					NS
*PAX5* ^neg^	36 (47%)	40 (53%)	41 (55%)	33 (45%)	
*PAX5* ^pos^	7 (47%)	8 (53%)	10 (77%)	3 (23%)	
**NEG B-ALL**					
	***RAG2*** **Expression**		***AID*** **Expression**		***p* ^†^**
	**Low** ***n*** ** = 22**	**High** ***n*** ** = 12**	**Low** ***n*** ** = 12**	**High** ***n*** ** = 21**	
**CNA**					*RAG2*: CNA^neg^ vs. CNA^pos^: 0.0464
CNA^neg^	9 (90%)	1 (10%)	3 (27%)	8 (73%)	
CNA^pos^	13 (54%)	11 (46%)	9 (41%)	13 (59%)	
1 CNA	4 (44%)	5 (56%)	2 (25%)	6 (75%)	
CNA^high^	9 (60%)	6 (40%)	7 (50%)	7 (50%)	
***IKZF1*** **deletion**					
*IKZF1* ^neg^	16 (80%)	4 (20%)	3 (15%)	17 (85%)	*RAG2*: 0.0257
*IKZF1* ^pos^	6 (43%)	8 (57%)	8 (67%)	4 (33%)	*AID*: 0.0029
***BCR::ABL1*^pos^** **B-ALL**					
	***RAG2*** **Expression**		***AID*** **Expression**		***p*** **^†^**
	**Low** ***n*** ** = 8**	**High** ***n*** ** = 21**	**Low** ***n*** ** = 22**	**High** ***n*** ** = 6**	
**CNA**					NS
CNA^neg^	3 (50%)	3 (50%)	4 (67%)	2 (33%)	
CNA^pos^	5 (22%)	18 (78%)	18 (82%)	4 (18%)	
1 CNA	1 (14%)	6 (86%)	5 (71%)	2 (29%)	
CNA^high^	4 (25%)	12 (75%)	13 (87%)	2 (13%)	
***IKZF1*** **deletion**					NS
*IKZF1* ^neg^	4 (40%)	6 (60%)	6 (60%)	4 (40%)	
*IKZF1* ^pos^	7 (27%)	19 (73%)	20 (80%)	5 (20%)	

^†^ computed by chi-squared or Fisher exact test. Abbreviations: *RAG2*, Recombination Activating Gene 2; *AID*, Activation Induced Cytidine Deaminase; CNA, Copy Number Alterations.

**Table 3:** Patients’ outcomes according to the primary aberration. (**A**) Univariate analysis of patients’ outcomes according to the primary aberration. (**B**) Multivariate analysis of patients’ outcomes according to the primary aberrations and demographic data.

(A)											
End Point and Variables	Total *n* = 161	NEG		*BCR::ABL1* *n* = 54	“Bad Primary”					*E2A::PBX* *n* = 3	*p*
		Total NEG * n = 61	NK Only *n* = 41		*BCR::ABL1*-like *n* = 15	*MLL::AF4* *n* = 5	Complex *n* = 15	Hyperdiploid *n* = 6	All “Bad Primary” ^‡^ *n* = 43		
**CR**											NEG vs. *BCR::ABL1*: 0.5454 ^†^
No. of patients	131/152	49/57	34/41	47/54	10/12	5/5	11/14	4/5	32/38	3/3	NEG vs. *BCR::ABL1-like*: 0.5555 ^†^
(%)	(86%)	(86%)	(83%)	(87%)	(83%)	(100%)	(79%)	(80%)	(78%)	(100%)	NEG vs. *“Bad”*: 0.5177 ^†^
**OS**											NEG vs. *BCR::ABL1*:0.0895 ^#^
No. of patients	161	61	41	54	15	5	15	6	43	3	NEG vs. *BCR::ABL1-like*: 0.1080 ^#^
4-year rate ± SE	34 ± 4%	32 ± 8%	25 ± 8%	54 ± 8%	10 ± 9%	53 ± 25%	20 ± 10%	0%	16 ± 7%	100%	**NEG vs. *“Bad”*: 0.0192 ^#^**
**RFS**											**NEG vs. *BCR::ABL1*: 0.0356 ^#^**
No. of patients	129	49	34	46	10	5	10	6	31	3	NEG vs. *BCR::ABL1-like*: 0.3400 ^#^
4-year rate ± SE	42 ± 6%	28 ± 9%	19 ± 9%	66 ± 8%	18 ± 16%	20 ± 18%	24 ± 15%	60 ± 22%	22 ± 10%	100%	**NEG vs. *“Bad”*: 0.0365 ^#^**
**DFS**											NEG vs. *BCR::ABL1*:0.0978 ^#^ NEG vs. *BCR::ABL1-like*: 0.5055 ^#^ **NEG vs. *“Bad”*: 0.0319 ^#^**
No. of patients	126	46	32	46	10	5	11	5	31	3	
4-year rate ± SE	37 ± 5%	25 ± 8%	18 ± 9%	58 ± 8%	16 ± 15%	20 ± 18%	22 ± 13%	40 ± 22%	17 ± 8%	100%	
**(B)**											
				**Primary Aberration**							
**End Point and Variables**	**Age**	**WBC**		**NEG** ***n*** ** = 61**	***BCR::ABL1*** ***n*** ** = 54**	**All “Bad Primary”** ***n*** ** = 43**	***BCR::ABL1-*** **like** ***n*** ** = 15**	***MLL::AF4*** ***n*** ** = 5**	**Complex** ***n*** ** = 15**	**Hyperdiploid** ***n*** ** = 6**	***E2A::PBX*** ***n*** ** = 3**
**CR**											
OR (95% CI)	**0.83 (0.70–0.98)**	1.08 (0.91–1.29)		1.00	0.99 (0.71–1.39)	0.90 (0.66–1.21)	0.92 (0.64–1.31)	1.07 (0.67–1.71)	0.86 (0.61–1.21)	0.86 (0.55–1.35)	1.11 (0.65–1.92)
*p* *	**0.0294**	0.3593		(reference)	0.9726	0.4782	0.6280	0.7738	0.3726	0.5156	0.6936
**OS**											
HR (95% CI)	**1.04 (1.03–1.06)**	1.00 (1.00–1.00)		1.00	**0.55 (0.32–0.96)**	1.67 (1.03–2.73)	1.39 (0.70–2.75)	1.10 (0.24–4.99)	1.62 (0.83–3.14)	3.91 (1.49–10.31)	2.62 (0.61–11.18)
*p* ^#^	**<0.0001**	0.8974		(reference)	**0.0338**	0.0261	0.3434	0.8984	0.1549	0.0058	0.1929
**RFS**											-
HR (95% CI)	**1.05 (1.03–1.07)**	1.00 (1.00–1.01)		1.00	**0.34 (0.16–0.72)**	1.81 (0.98–3.37)	1.21 (0.49–3.02)	**4.40 (1.37–14.13)**	1.72 (0.71–4.14)	3.61 (0.81–16.18)	
*p* ^#^	**<0.0001**	0.0929		(reference)	**0.0049**	0.0597	0.6782	**0.0129**	0.2259	0.0934	

(A) * NEG includes patients negative for all molecular markers with NK, or other aberrations of unknown significance, or without cytogenetic data. ^‡^ Two hypodiploid cases are included in all the “bad genetics” groups but are not included in the preceding subgroups. ^†^ computed by Fisher’s exact test. ^#^ computed by the log-rank test. Abbreviations: NK, normal karyotype; CR, complete remission; OS, overall survival; RFS, relapse-free survival; DFS, disease-free survival; SE, standard error. (B) * computed from General Regression Model. ^#^ computed by Cox proportional hazard regression. Abbreviations: WBC, white blood count; CR, complete remission; OS, overall survival; RFS, relapse-free survival; OR, odds ratio; HR, hazard ratio; 95% CI, 95% confidence interval.

**Table 4:** Patients’ outcomes according to the secondary aberrations. (**A**) Univariate analysis of patients’ outcomes according to the secondary aberrations. (**B**) Multivariate analysis of patients’ outcomes according to the secondary aberrations, adjusted for age and WBC.

(A)																				
End Point and Variables	Total	CNA Presence				CNA Mutation Burden				*IKZF1* Mutation Status				Bad CNA (CNA^high^/*IKZF*^pos^)				Good CNA (CNA^pos^ Other than Bad CNA)		
		CNA^neg^	CNA^pos^	*p*		CNA^low^	CNA^high^	*p*		*IKZF* ^neg^	*IKZF* ^pos^	*p*		CNA^high^ *IKZF*^pos^	All Other Patients	*p*		Good CNA	All Other Patients	*p*
**TOTAL B-ALL**																				
**CR**				0.5283 ^†^				**0.0422** ^†^				0.3746 ^†^				0.0514 ^†^				0.0880 ^†^
**No. of patients**	73/88	22/26	51/62			45/50	28/38			41/48	38/47			20/28	53/60			31/34	42/54	
**(%)**	83%	85%	82%			90%	74%			85%	85%			71%	88%			91%	78%	
**OS**				0.4921 ^#^				**0.0179** ^#^				0.2734 ^#^				**0.0045** ^#^				**0.0427** ^#^
No. of patients	92	27	65			52	40			52	47			28	64			37	55	
4-year rate ± SE	36 ± 6%	43 ± 10%	28 ± 6%			42 ± 8%	20 ± 7%			41 ± 8%	26 ± 7%			9 ± 6%	42 ± 7%			43 ± 9%	25 ± 6%	
**RFS**				0.1460 ^#^				**0.0412** ^#^				0.0320 ^#^				**0.0035** ^#^				0.2096 ^#^
No. of patients	73	22	49			44	27			40	37			19	52			30	41	
4-year rate ± SE	41 ± 7%	43 ± 10%	34 ± 8%			53 ± 9%	20 ± 10%			48 ± 10%	31 ± 9%			14 ± 9%	49 ± 8%			45 ± 10%	40 ± 9%	
**NEG B-ALL**																				
**CR**				0.6317 ^†^				0.1710 ^†^				0.5419 ^†^				0.3105 ^†^				0.4581 ^†^
No. of patients	28/32	9/10	19/22			18/19	10/13			17/19	11/13			7/9	21/23			12/13	16/19	
(%)	88%	90%	86%			95%	77%			89%	85%			78%	91%			92%	84%	
**OS**				0.2117 ^#^				**0.0016** ^#^				**0.0055** ^#^				**0.0051** ^#^				0.1513 ^#^
No. of patients	34	10	24			19	15			20	14			10	24			14	20	
4-year rate ± SE	28 ± 9%	53 ± 17%	22 ± 9%			45 ± 15%	7 ± 6%			46 ± 13%	7 ± 7%			0%	40 ± 12%			28 ± 13%	25 ± 10%	
**RFS**				0.3322 ^#^				**0.0171** ^#^				**0.0118** ^#^				**0.0037** ^#^				0.1042 ^#^
No. of patients	28	9	19			18	10			17	11			7	21			12	16	
4-year rate ± SE	25 ± 10%	42 ± 20%	19 ± 11%			43 ± 14%	0%			36 ± 15%	10 ± 10%			0%	34 ± 14%			31 ± 17%	22 ± 12%	
***BCR::ABL1*** **^pos^** ** B-ALL**																				
**CR**				0.3438 ^†^				0.3954 ^†^				0.5441 ^†^				0.2613 ^†^				0.0653 ^†^
No. of patients	24/30	4/6	20/24			12/14	12/16			8/10	22/26			10/14	14/16			10/10	14/20	
(%)	80%	67%	83%			86%	75%			80%	85%			71%	88%			100%	70%	
**OS**				0.3019 ^#^				0.2129 ^#^				0.6468 ^#^				**0.0406** ^#^				**0.0025** ^#^
No. of patients	29	6	23			14	15			10	25			13	16			10	19	
4-year rate ± SE	47 ± 10%	33 ± 19%	51 ± 11%			62 ± 13%	33 ± 13%			60 ± 15%	45 ± 11%			20 ± 12%	67 ± 12%			89 ± 10%	25 ± 11%	
**RFS**				0.8073 ^#^				0.1157 ^#^				0.0875 ^#^				**0.0231** ^#^				**0.0404** ^#^
No. of patients	23	4	19			12	11			8	21			9	14			10	13	
4-year rate ± SE	65 ± 11%	75 ± 22%	63 ± 12%			82 ± 12%	44 ± 17%			88 ± 12%	48 ± 12%			29 ± 17%	85 ± 10%			89 ± 10%	44 ± 15%	
**(B)**																				
**End Point and Variables**				**CNA^high^**					***IKZF*** **^pos^**				**Bad CNA** **(CNA^high^/*IKZF*^pos^)**				**Good CNA** **(CNA^pos^ Other than Bad CNA)**			
**TOTAL B-ALL**																				
**CR**																				
**OR (95% CI)**				0.83 (0.67–1.02)					0.97 (0.78–1.21)				0.83 (0.67–1.04)				1.20 (0.97–1.48)			
***p* ** *				0.0791					0.8156				0.1054				0.0952			
**OS**																				
HR (95% CI)				1.81 (1.04–3.17)					0.91 (0.51–1.61)				1.92 (1.06–3.48)				0.56 (0.31–1.01)			
*p* ^‡^				**0.0374**					0.7393				**0.0318**				0.0521			
**RFS**																				
HR (95% CI)				1.97 (0.99–3.92)					1.48 (0.74–2.97)				2.89 (1.37–6.10)				0.57 (0.28–1.16)			
*p* ^‡^				0.0538					0.2695				**0.0054**				0.1189			
**NEG B-ALL**																				
**CR**																				
OR (95% CI)				0.68 (0.47–0.98)					0.87 (0.58–1.31)				0.74 (0.50–1.10)				1.15 (0.77–1.71)			
*p* *				**0.0397**					0.4921				0.1305				0.4897			
**OS**																				
HR (95% CI)				4.95 (1.70–14.39)					4.12 (1.37–12.33)				4.85 (1.56–15.09)				0.42 (0.14–1.20)			
*p* ^‡^				**0.0033**					**0.0114**				**0.0065**				0.1050			
**RFS**																				
HR (95% CI)				4.11 (1.30–12.99)					4.59 (1.32–15.97)				9.80 (2.32–41.35)				0.32 (0.09–1.08)			
*p* ^‡^				**0.0160**					**0.0165**				**0.0019**				0.0660			
***BCR:ABL1*^pos^** **B-ALL**																				
**CR**																				
OR (95% CI)				0.97 (0.68–1.40)					1.16 (0.84–1.62)				0.94 (0.65–1.38)				1.28 (0.88–1.87)			
*p* *				0.8860					0.3595				0.7485				0.1871			
**OS**																				
HR (95% CI)				1.84 (0.60–5.63)					0.88 (0.27–2.91)				2.36 (0.76–7.27)				0.11 (0.01–0.90)			
*p* ^‡^				0.2860					0.8326				0.1359				**0.0399**			
**RFS**																				
HR (95% CI)				6.29 (0.89–44.47)					3.52 (0.40–31.35)				7.65 (1.21–48.31)				0.14 (0.02–1.22)			
*p* ^‡^				0.0654					0.2564				**0.0304**				0.0756			

^†^ computed by chi-squared or Fisher’s exact test. ^#^ computed by log-rank test. * computed from General Linear Model. ^‡^ computed by Cox proportional hazard regression. Abbreviations: WBC, white blood count; CR, complete remission; OS, overall survival; RFS, relapse-free survival; SE, standard error; CNA, copy number alteration; HR, hazard ratio; OR, odds ratio; 95% CI, 95% confidence interval.

**Table 5:** Outcome of B-ALL patients according to the revised code for risk stratification.

End Point and Variables	Intermediate Risk	Low Risk	High Risk	Total
**CR**				
No. of patients	24/28	13/13	44/55	81/96
(%)	86%	100%	80%	85%
*p* *	(reference)	0.1514	0.5224	
**OS**				
No. of patients	29	13	58	100
4-year rate ± SE	41 ± 10%	75 ± 13%	13 ± 5%	30 ± 5%
*p* ^‡^	(reference)	**0.0148**	**0.0011**	
**RFS**				
No. of patients	26	13	42	81
4-year rate ± SE	39 ± 12%	90 ± 9%	23 ± 7%	38 ± 7%
*p* ^‡^	(reference)	**0.0254**	**0.0037**	
**DFS**				
No. of patients	24	13	43	80
4-year rate ± SE	36 ± 11%	90 ± 9%	17 ± 6%	34 ± 6%
*p* ^‡^	(reference)	**0.0183**	**0.0010**	
**Multivariate analysis adjusted for age and WBC**				
**CR**				
OR (95% CI)	1.00	0.92 (0.73–1.16)	1.12 (0.89–1.40)	
*p* ^†^	(reference)	0.3259	0.4713	
**OS**				
HR (95% CI)	1.00	0.13 (0.02–0.99)	2.12 (1.14–3.93)	
*p* ^#^	(reference)	**0.0484**	**0.0176**	
**RFS**				
HR (95% CI)	1.00	0.16 (0.02–1.23)	2.59 (1.25–5.33)	
*p* ^#^	(reference)	0.0787	**0.0100**	

* computed from the chi-squared test. ^‡^ computed from log-rank test. ^†^ computed from a General Linear Model. ^#^ computed by Cox proportional hazard regression. All *p*-values are calculated as a comparison to the intermediate risk group. Abbreviations: CR, complete remission; OS, overall survival; RFS, relapse-free survival; DFS, disease-free survival; SE, standard error; HR, hazard ratio; OR, odds ratio; 95% CI, 95% confidence interval. Intermediate risk: *BCR::ABL1*^pos^: CNA^neg^; NEG: CNA^neg^ or 1 CNA/*IKZF1*^pos^ or CNA^pos^/*IKZF1*^neg^. Low risk: *E2A::PBX*^pos^; *BCR::ABL1*^pos^: 1 CNA/*IKZF1*^pos^ or CNA^pos^/*IKZF1*^neg^. High risk: *MLL::AF4*^pos^; Complex; Hyperdiploid; Hypodiploid; *BCR::ABL1*-like; CNA^high^/*IKZF1*^pos.^

## Data Availability

The datasets used and analyzed during the current study are available from the corresponding author on reasonable request.

## Supplementary Materials

The following supporting information can be downloaded at: https://www.mdpi.com/article/10.3390/cancers15225431/s1, Table S1: Characteristics of detected markers or surrogates of *BCR::ABL1*-like B-ALL; Table S2: (A) AID/RAG expression signatures in correlation with the primary aberrations; (B) AID/RAG expression signatures in correlation with the distribution of CNA profiles; Table S3: (A) Frequency of CNA mutation burden and specific gene deletions in genetic subgroups of the studied cohort; (B) Correlation between CNA mutation burden and deletions in each gene; Table S4: Univariate analysis of patients’ outcome in subgroups according to CNA status; Table S5: (A) Frequency of patients with relapse and those remaining in remission—in subgroups according to mutator enzyme expression; (B) Univariate analysis of CNA^neg^ patients’ outcomes according to mutator enzyme expression; (C) Multivariate analysis of CNA^neg^ patients’ outcomes according to mutator enzyme expression, adjusted for age and WBC; Figure S1: Outcome of *IKZF1*-positive patients according to the number of CNAs. Kaplan-Meier estimates for the probability of overall survival and relapse-free survival in *IKZF1*-positive patients according to the number of copy number alterations (CNAs). CNA^high^—≥2 detected CNAs. (A,B)—overall survival and relapse-free survival in all *IKZF1*-mutated patients. (C,D)—overall survival and relapse-free survival in *BCR::ABL1*-positive *IKZF1*-positive patients only. *n*—number of patients, *p*—p-value; Figure S2: Outcome of CNA^high^ patients according to *IKZF1* mutation status. Kaplan-Meier estimates for the probability of (A) overall survival and (B) relapse-free survival in CNA^high^ patients according to *IKZF1* mutation status. CNA^high^—≥2 detected CNAs. *n*—number of patients, *p*—*p*-value; Figure S3: Outcome of CNA^high^ patients according to *IKZF1* and *CDKN2A/B* mutation status. Kaplan-Meier estimates for the probability of (A) overall survival and (B) relapse-free survival in CNA^high^ *CDKN2A/B^pos^* patients according to *IKZF1* mutation status. The graph only contains *CDKN2A/B*^pos^ patients. *n*—number of patients, *p*—*p*-value; Figure S4: Outcome of CNA^neg^ patients according to *RAG2* and *AID* expression. Kaplan-Meier estimates for the probability of overall survival and relapse-free survival in CNA^neg^ patients according to the *AID* and *RAG2* expression levels. (A,B)—overall survival and relapse-free survival in CNA^neg^ patients according to the *AID* expression level. (C,D)—overall survival and relapse-free survival in CNA^neg^ patients according to the *RAG2* expression level. CNA^neg^—0 detected copy number alterations, *n*—number of patients, *p*—*p*-value; Figure S5: Outcome of CNA^pos^ patients according to *RAG2* and *AID* expression. Kaplan-Meier estimates for the probability of overall survival and relapse-free survival in CNA^pos^ patients according to the *AID* and *RAG2* expression levels. (A,B)—overall survival and relapse-free survival in NEG CNA^pos^ patients according to the *AID* expression level. (C,D)—overall survival and relapse-free survival in *BCR::ABL1*^pos^ CNA^pos^ patients according to the *RAG2* and *AID* expression levels. CNA^pos^—at least 1 detected copy number alteration, *n*—number of patients, *p*—*p*-value; Figure S6: Outcome of younger and older patients according to CNA profile. Kaplan-Meier estimates for the probability of overall survival and relapse-free survival in all studied patients according to the CNA profile, divided into younger (≤40 y.) and older (>40 y.) patients. (A,B)—overall survival and relapse-free survival in younger patients, grouped into CNA^high^ / *IKZF1*^pos^ and all other CNA profiles. (C,D)—overall survival and relapse-free survival in older patients, grouped into CNA^high^ / *IKZF1*^pos^ and all other CNA profiles. CNA^high^—at least 2 detected copy number alteration, *n*—number of patients, *p*—*p*-value. References [44,46,67,68,69,70,71,72] are cited in the supplementary materials.
