# Supplementary material for: Prognostic Impact of Copy Number Alterations’ Profile and AID/RAG Signatures in Acute Lymphoblastic Leukemia (ALL) with BCR::ABL and without Recurrent Genetic Aberrations (NEG ALL) Treated with Intensive Chemotherapy
Source: Cancers (Basel). 2023 Nov 15;15(22):5431. doi: 10.3390/cancers15225431 (PMC10670434; doi:10.3390/cancers15225431)
Supplement: Supplementary file 1 [file cancers-15-05431-s001.zip › cancers-2681173-supplementary.pdf]

# Supplementary Materials

## Prognostic Impact of Copy Number Alterations' Profile and AID/RAG Signatures in Acute Lymphoblastic Leukemia (ALL) with BCR::ABL and without Recurrent Genetic Aberrations (NEG ALL) Treated with Intensive Chemotherapy

## Supplementary methods

### *Genetic material collection and preparation*

Pretreatment bone marrow and/or peripheral blood specimens were enriched in mononuclear cells by Ficoll density gradient centrifugation. Genomic DNA was extracted from cryopreserved mononuclear cells by using the DNAzol reagent (Invitrogen, Karlsruhe, Germany) and total RNA was isolated using the Trizol reagent (Invitrogen, Karlsruhe, Germany), according to the manufacturer's instructions.

### *Molecular testing:*

### *Molecular markers and surrogates of BCR::ABL1-like phenotype*

RT-PCR was used to identify cases positive for aberrations activating ABL and JAK-STAT pathways: *P2RY8-CRLF2, ZMIZ1-ABL1, RCSD1-ABL1, RANBP2-ABL1, NUP214-ABL1, ETV6-ABL1, ZC3HAV1-ABL2, RCSD1-ABL2, PAG1-ABL2, SSBP2-PDGFRB, ZEB2-PDGFRB, TNIP1-PDGFRB, EBF1-PDGFRB, STRN3-JAK2, PAX5-JAK2, ETV6-JAK2, EBF1-JAK2, BCR-JAK2*[1,2]. Genomic PCR was used to identify point mutations in *JAK1* (exon 14), *JAK2* (exons 10-19), *CRLF2* (exon 6), *IL7R* (exon 6) genes [1,3–5]. Then PCR products were directly sequenced by an ABI3730xl DNA Sequencer (Applied Biosystem, Foster City, CA, USA).

*CRLF2* overexpression were assessed by qPCR as the surrogate of *BCR::ABL1-like* profile. It was calculated using the ΔΔCp method, where ΔΔCp = ($CRLF2_{sample}$-$GUS_{sample}$) - ($CRLF2_{calibrator}$-$GUS_{calibrator}$) and the relative expression level of *CRLF2* is $2^{-\Delta\Delta Cp}$. As a calibrator cDNA obtained from healthy volunteer mononuclear cells[6]. The $2^{-\Delta\Delta Ct}$ values for *CRLF2* overexpression was established >1000.

### *Gene expression by real-time polymerase chain reaction*

Expression was evaluated using the TaqMan Gene Expression assay Hs01851142_s1 for *RAG2* and Hs00757808_m1 for *AID* (Life Technologies, Carlsbad, CA, USA).The expression of both genes was calculated in a single sample using the relative expression level ΔCp method (where ΔCp = *RAG2* or $AID_{sample}$-$GUS_{sample}$). Patients were divided into 2 subgroups according to median of relative expression as the threshold. In this manuscript we refer these subgroups as high vs low *RAG2* and *AID* cases.

Quality of RNA material was evaluated using housekeeping gene GUS by quantitative real-time polymerase chain reaction (qPCR) on a LightCycler 480 II (Roche Applied Science, Basel, Switzerland) [7]. Samples with Cp GUS values above 32 were found to be degraded RNA and withdrawn from the study.

### *Multiplex Ligation-Dependent Probe Amplification (MLPA)*

DNA was subjected to multiplex ligation-dependent probe amplification (MLPA) reaction using the SALSA MLPA P335-B2 ALL-IKZF1 (MRC-Holland, Amsterdam, the Netherlands) kit according to the manufacturer's instructions. This kit included probes for detection of deletions of IKAROS family zinc finger 1 (*IKZF1*), purinergic receptor P2Y8 (*P2RY8*), zinc finger protein, Y-linked (ZFY), Janus kinase 2 (*JAK2*), paired box 5 (*PAX5*), ETS variant 6 (*ETV6*), RB transcriptional corepressor 1 (*RB1*), BTG anti-proliferation factor 1 (*BTG1*), early B-cell factor 1 (*EBF1*), cyclin dependent kinase inhibitor 2A/2B (*CDKN2A/2B*), cytokine receptor like factor 2 (*CRLF2*), interleukin 3 receptor subunit $\alpha$ (*IL3RA*), colony-stimulating factor 2 receptor $\alpha$ subunit (*CSF2RA*) and short stature homeobox (*SHOX*) genes. Electrophoresis were performed on ABI3730xl DNA Sequencer (Applied Biosystem, Foster City, CA, USA). The resulting peak intensities were normalized to the manufacturer's control probes to calculate the relative copy number and the DNA from the healthy control was used as a reference.Dosage

quotient (DQ) values between 0,75 and 1,3 were considered normal copy number of 2, while any value above or below this threshold was scored as gain or loss. For *CDKN2A/B*, deletion of either locus was considered as deleted.

***Evaluation of IKZF1 mRNA isoforms (Δ3-6, Δ1-7) by RT-PCR***

Additionally, the whole series (163 patients) was evaluated for expression of 2 isoforms of *IKZF1* mRNA (Δ3-6, Δ1-7) according to [8]. Obtained data were correlated with MLPA analysis and positive results were incorporated for survival analysis according to *IKZF1* status (49 *IKZF1$^{pos}$* cases referred as "MLPA+RT-PCR group" – see details in Table 1C). In order to evaluate patients' characteristics, only *IKZF1$^{pos}$* cases from the group of 94 patients for whom the MLPA analysis of other gene deletions was performed - were included (39 *IKZF1$^{pos}$* cases referred as "MLPA group": 37 cases identified using MLPA, and 2 cases using RT-PCR – see details in Table 1C).

***Treatment protocols***

According to the protocol, the pre-treatment for patients with ALL without Philadelphia chromosome/*BCR::ABL1* (*BCR::ABL1*$^{neg}$ ALL) incorporated prednisone and intrathecal prophylaxis with triple therapy (cytarabine, methotrexate, dexamethasone) or liposomal cytarabine. The first induction was uniform for all patients with *BCR::ABL1*$^{neg}$ ALL aged up to 55 and comprised prednisone, daunorubicin, peg-asparaginase, and vincristine. Alongside systemic chemotherapy, patients received intrathecal triple therapy or liposomal cytarabine with each block of chemotherapy. Upon completion of the first induction, the state of hematological remission and MRD by MFC were evaluated. Patients achieving CR with MRD below 0.1% progressed directly to the consolidation phase. Those who, post-first induction, achieved remission but had MRD at a level of 0.1% or higher, underwent a second induction according to the FLAM program (fludarabine, cytarabine, and mitoxantrone) or miniFLAM (reduced number of cytostatic doses), contingent on age (respectively: up to 40 years and older). Additionally, patients with CD52 positive disease could receive alemtuzumab. After the second induction of remission, the state of hematological remission and MRD were reevaluated. Patients in the CR phase then proceeded to the consolidation phase of remission or underwent alloHSCT. For patients over the age of 55, lower doses of daunorubicin were administered, and dexamethasone was used as a substitute for prednisone. In this patient group, monitoring MRD did not influence therapeutic decisions during induction as all received a second induction cycle comprising cyclophosphamide, cytarabine, and mercaptopurine.

Under the PALG ALL6 protocol, *BCR::ABL1*$^{neg}$ patients up to 55 years old achieving complete remission after one or two induction cycles were prescribed two consolidation cycles. The first consolidation comprised intermediate doses of methotrexate, combined with etoposide and dexamethasone, while the second involved high doses of cytarabine along with cyclophosphamide and peg-asparaginase. The state of remission and MRD were assessed after each cycle.

Post the second consolidation cycle, risk stratification was determined based on the assessment of MRD status at different treatment stages. Patients classified under the standard risk group (MRD below 0.1% post the first induction cycle, and below 0.01% post the first and second consolidation blocks), who didn't undergo alloHSCT, received a third consolidation cycle. Those assigned to the high-risk group due to higher MRD levels or delayed CR proceeded to alloHSCT. Individuals over the age of 55 underwent two or three consolidation cycles, with a reduced dose intensity compared to younger patients.

For *BCR::ABL1*$^{pos}$ ALL patients up to 55 years old, the PALG ALL6 protocol required the administration of imatinib along with vincristine, daunorubicin, and dexamethasone. Following induction completion, the state of remission and molecular MRD were evaluated. If hematologic remission was not achieved post-induction, dasatinib was incorporated into the subsequent treatment. Additionally, patients ineligible for alloHSCT, with positive MRD, had imatinib switched to dasatinib, with the concurrent administration of dexamethasone recommended during the initial four weeks of dasatinib treatment.

Upon achieving CR, alloHSCT was performed post-induction if MRD was below 0.05% or post the second consolidation.

For *BCR::ABL1*$^{pos}$ ALL patients over 55, the PALG protocol, analogous to the younger group, incorporated the use of imatinib. In cases of primary or secondary resistance to imatinib, dasatinib was administered. Reduced-intensity conditioning alloHSCT was also considered for each patient following the second consolidation cycle. For those not candidates for alloHSCT, maintenance therapy utilizing TKI monotherapy ensued after six consolidation cycles, continuing until progression or until alloHSCT is feasible.

## Supplementary results

***Clinical and biological characteristics of RAG2 and AID mutator enzymes' expression – in correlation with primary genetic subgroups – continued from main manuscript.***

Patients with signature 2 with *AID* abundance, and 3 with *RAG2* abundance (64% of the whole series) showed an inverse correlation of both enzymes with each other (p=0.0004; see Table S2). The distribution of *RAG2* and *AID* expression signatures across genetic subgroups and correlation with other clinical and biological data are presented in Table 1B and Table S2.

The frequent prevalence of high *AID* expression cases was associated with preB immunophenotype (36% vs. 64%, P=0.03). Interestingly, older patients (≥37 years old) presented a higher frequency of high *AID* expression compared to the younger ones (41% vs. 59%; P=0.017).

***Clinical and biological characteristics of secondary CNA aberrations– in correlation with primary genetic subgroups – continued from main manuscript.***

Due to the limited access to the high-quality DNA material for CNA detection, we were able to perform the analysis only for 94 patients. The detailed patients' characteristics according to CNAs are shown in Table 1C. Within the CNA$^{pos}$ population, a single CNA was detected in 25 out of 94 studied patients (26.5%), double CNAs were found in 22/94 (23.4%) patients, while 19 out of 94 patients harbored ≥3 CNAs (20%), which represented the highest level of genetic instability (Table S3).

The frequencies of different deleted genes are shown in Figure 1B and Table S3: the most frequent deletion was found in the *IKZF1* gene (41/94; 43.6% of total B-ALL), followed by *CDKN2A/B* (32/94; 33%), *PAX5* (15/94; 16%), *EBV6* and *EBF1* (both 14/94; 15%) in the whole cohort. When looking into different genetic subgroups, *IKZF1*$^{pos}$ CNA was the most frequent deletion in *BCR::ABL*, *BCR::ABL*-like followed by NEG subpopulations (67%, 50%, and 40%, respectively; see Table S3A). Moreover, *IKZF1* deletions were most frequently observed in the ≥3 CNA category (95%), followed by *CDKN2A/B* and *PAX5* deletions (63% and 58% respectively; see details in Table S3B).

Finally, CNA$^{pos}$ patients were older when compared to the CNA$^{neg}$ population (median of 33y. vs. 39.5y. for CNA$^{neg}$ vs CNA$^{pos}$ population, respectively; p=0.023), and the age was increasing with the number of CNAs: 40.1y. for 1CNA vs 41.3y. for 2CNAs vs 43.7y. for ≥3 CNAs category. In the same manner, the WBC level increased with the number of CNAs: 24.56 × $10^9$/L for 1CNA vs 48.71 × $10^9$/L for ≥3 CNAs (p=0.052); see Table 1C for details). Interestingly, *IKZF1*deletions presented specific clinico-biological features, distinguishing them from other CNA$^{pos}$ patients lacking *IKZF1* deletions: an older age (41.0y. vs 35.0y.) as well as a higher WBC (19.0 ×$10^9$/L vs 13.8 ×$10^9$/L).

***Correlation of secondary CNA aberrations with RAG2 /AID signatures - continued from main manuscript.***

The data on a functional correlation of the *AID/RAG2* expression signatures with CNAs' profiles are presented in Table 2 and Table S2. Briefly, we found a clear association between CNA *IKZF1*$^{pos}$ and a high *RAG2* expression only (72% of *IKZF1*$^{pos}$ CNAs in *AID*$^{low}$/*RAG2*$^{high}$), particularly in *BCR::ABL1*$^{pos}$ context (p=0.001). Interestingly, not a single CNA *IKZF1*$^{pos}$ patient from the *BCR::ABL1*$^{pos}$ subgroup was

accompanied by a high *AID* expression without *RAG2* abundance (0/18 in *AID*$^{high}$/*RAG2*$^{low}$; p<0,001). This pattern was even more pronounced for *IKZF1*$^{pos}$ cases when accompanied by other gene deletions (CNA$^{high}$/*IKZF1*$^{pos}$), that were absent in *AID*$^{high}$/*RAG2*$^{low}$ subset in the whole series (0/26) when compared to 13/26 (50%) accompanied by *AID*$^{low}$/*RAG2*$^{high}$ (p<0,001). On the contrary, CNA$^{neg}$ or other CNAs lacking *IKZF1* correlated positively with *AID*$^{high}$/*RAG2*$^{low}$ signature, particularly in NEG ALL context (70%, 7/10 CNA$^{neg}$; and 66%, 6/9 other CNA$^{pos}$ *IKZF1*$^{neg}$).

***Prognostic impact of the number of copy number alterations (CNA) and CNAs' mutation burden on patients' outcome.***

We compared survival according to the CNA status. However, no significant differences were observed between CNA$^{pos}$ and CNA$^{neg}$ in genetic subgroups, as well as in the whole series (Table 4A; p=0.53 for total series). As the CNA$^{pos}$ subgroup was heterogeneous with respect to the mutation burden and the gene type, we stratified patients according to the number of CNAs and found that patients with a single CNA (1CNA) had a clearly better survival than patients with 2 and more CNAs (Table S4; p=0.014). Importantly, no differences in prognostic impact were noted between the individual genes within the 1CNA group. Additionally, there was no significant difference in survival between CNA$^{neg}$ and 1CNA patients (Table S4; p=0.51). Thus, in order to look for correlations, we grouped the patients according to genetic instability level: the 1$^{st}$ subgroup was labeled as "CNA$^{low}$" and included both CNA$^{neg}$ and 1CNA patients, while the 2$^{nd}$ subgroup was described as "CNA$^{high}$" and included patients who harbored ≥2CNAs (see Table 1C for details).

When we compared the outcome of these 2 subgroups, we observed significant differences in survival in the NEG cohort as well as in the whole series: patients with CNA$^{high}$ had lower OS and RFS probabilities than CNA$^{low}$ patients (OS: 7%vs. 45%, p=0.002; RFS: 0% vs. 43%, p=0.017, respectively, in NEG B-ALL; see Figure 3 and Table 4A).

***IKZF1 deletions correlates with poor outcome in the whole series, while IKZF1 CNA coexisting with other CNAs present worst outcome in IKZF1$^{pos}$ subpopulation***

*IKZF1* was the most frequent deletion in our study cohort, and it significantly correlated with lower OS and RFS in the NEG B-ALL subgroup (OS 7% vs. 46%, p=0.006; RFS: 10% vs. 36%, p=0.012 for *IKZF1*$^{pos}$ vs *IKZF1*$^{neg}$ respectively), and with lower RFS in the whole series (RFS: 31% vs. 48%, p=0.032 for *IKZF1*$^{pos}$ vs *IKZF1*$^{neg}$ respectively; see Figure 4 and Table 4A for details). A trend towards statistical significance was observed in the *BCR::ABL1*$^{pos}$ cohort (RFS, p=0.087).

The CNA$^{high}$/*IKZF1*$^{pos}$ patients showed worse survival when compared to 1CNA/*IKZF1*$^{pos}$ series and this was valid in the *BCR::ABL1*$^{pos}$ cohort (OS: 20% vs 80% respectively; p=0.027; RFS: 29% vs 80% respectively; p=0.088), and in the whole series (OS: 9% vs 56% respectively; p=0.001; RFS: 14% vs 52% respectively; p=0.039; Figure S1 and Table S4). Whereas, only a trend towards significance was found in the NEG subpopulation, probably due to a small number of 1CNA/*IKZF1*$^{pos}$ patients (Table S4).

***CNA$^{high}$/IKZF1$^{pos}$ patients display inferior survival when compared to CNA$^{high}$/IKZF1$^{neg}$ series***

When we compared the outcome of CNA$^{high}$/*IKZF1*$^{pos}$ vs CNA$^{high}$/*IKZF1*$^{neg}$ series, we found that the presence of *IKZF1* deletions conferred lower survival RFS in the NEG ALL (OS 0% vs 20% respectively; p=0.08; RFS 0% vs 49% respectively; p=0.007), OS in the *BCR::ABL1*$^{pos}$ cohort (OS 20% vs 100% respectively; p=0.035; RFS 29% vs 100% respectively; p=0.07) and OS and RFS in the whole ALL series (OS 9% vs 42% respectively; p=0.033; RFS 14% vs 39% respectively; p=0.003; Table S4 and Figure S2). These data suggest again, that the negative outcome observed in the total CNA$^{high}$ population may be mostly attributed to patients with CNA$^{high}$/*IKZF1*$^{pos}$.

***CDKN2A/B deletions accompanied by IKZF1 CNA present worse outcome when compared to CNA$^{high}$/CDKN2A/B$^{pos}$/IKZF1$^{neg}$ series***

Since some studies reported that *CDKN2A/B* deletions correlated with worse outcome when accompanied by other deletions, we compared the impact of *IKZF1* deletions in the

CNA$^{high}$/*CDKN2A/B*$^{pos}$ cohort, and found that *CDKN2A/B*$^{pos}$ patients accompanied by *IKZF1* deletion showed worse outcomes when compared to the subgroup without *IKZF1* (OS 8% vs 35% respectively; p=0.012; RFS 12% vs 40%; p=0.026 for whole series; Figure S3). Despite the relatively small number of patients, this effect was particularly evident in the *BCR::ABL1*$^{pos}$ series (p=0.01). Additionally, there was no difference between CNA$^{high}$/*IKZF1*$^{pos}$ accompanied by *CDKN2A/B* deletion and other CNA$^{high}$/*IKZF1*$^{pos}$ without *CDKN2A/B* deletion, suggesting that coexistence of a *CDKN2A/B* gene deletion alone may not affect the outcome of the CNA$^{high}$ population. Furthermore, *CDKN2A/B* deletions with coexisting *IKZF1* deletions shared the same *AID*$^{low}$/*RAG2*$^{high}$ expression signature as other *IKZF1*$^{pos}$ CNAs (Table S2). On the other hand, CNA$^{high}$/*CDKN2A/B*$^{pos}$/*IKZF1*$^{neg}$ did not display this characteristic, suggesting a different mutational mechanism.

***Prognostic impact of the dominant negative Ik6 isoform of IKZF1 mutation within poor-risk CNA$^{high}$/IKZF1$^{pos}$ subgroup***

Dominant negative Ik6 isoform of *IKZF1* deletion was described in other studies to correlate with the worst outcome. Indeed, despite a small group of patients, we found strong negative impact of Ik6 on relapse rate among the poor-risk CNA$^{high}$/*IKZF1*$^{pos}$ subgroup (RFS 0% vs 28% Ik6 vs other *IKZF* deletions, respectively, in the CNA$^{high}$/*IKZF1*$^{pos}$ subpopulation; p=0.034). In contrast, patients with Ik6 did not show any difference in survival in the 1CNA/*IKZF1*$^{pos}$ population.

## Supplementary tables

**Table S1.** Characteristics of detected markers or surrogates of *BCR::ABL1*-like B-ALL.

| | Gene | Exon | Abberation | Age [years] | WBC [x $10^9$/L] | Immunological subtype |
|---|---|---|---|---|---|---|
| 1 | *JAK2*<br>*CRLF2* | 16<br>-. | c.2047 A>G; p.R683G<br>overexpression ($2^{-\Delta\Delta Ct}$=5442) | 45 | 233.9 | common |
| 2 | *JAK2*<br>*CRLF2* | 16<br>- | c.2047 A>G; p.R683G<br>overexpression ($2^{-\Delta\Delta Ct}$=13777) | 18 | 60.49 | common |
| 3 | *CRLF2* | - | *P2RY8-CRLF2* | 48 | 12.9 | common |
| 4 | *CRLF2* | - | *P2RY8-CRLF2* | 41 | 3.9 | common |
| 5 | *CRLF2* | - | *P2RY8-CRLF2* | 40 | 44,5 | prepreB |
| 6 | *CRLF2* | - | *P2RY8-CRLF2* | 43 | 3 | common |
| 7 | *CRLF2* | - | *P2RY8-CRLF2* | 64 | 1,34 | common |
| 8 | *CRLF2* | - | overexpression ($2^{-\Delta\Delta Ct}$=4770) | 69 | 157 | common |
| 9 | *JAK2*<br>*CRLF2* | 16<br>- | c.2047 A>G; p.R683G<br>overexpression ($2^{-\Delta\Delta Ct}$=2120) | 37 | 60 | common |
| 10 | *CRLF2* | - | overexpression ($2^{-\Delta\Delta Ct}$=1478) | 49 | 1,3 | common |

| | | | | | | |
|---|---|---|---|---|---|---|
| 11 | *CRLF2* | - | *P2RY8-CRLF2* | 19 | 11.78 | common |
| 12 | *CRLF2* | - | overexpression ($2^{-\Delta\Delta Ct}$=9216) | 44 | 121 | common |
| 13 | *JAK2*<br>*CRLF2*<br>*CRLF2* | 16<br>6<br>-. | c.2047 A>G; p.R683G<br>c.695 T>A; p.F232C<br>overexpression ($2^{-\Delta\Delta Ct}$=248333) | 54 | 68.9 | preB |
| 14 | *CRLF2* | - | *P2RY8-CRLF2* | 34 | 55.8 | preB |
| 15 | *JAK2*<br>*CRLF2* | 16<br>- | c.2047 A>G; p.R683G<br>overexpression ($2^{-\Delta\Delta Ct}$=22073) | 19 | 11.3 | preB |
| 16 | *JAK2* | 20 | c.2705 T>A; p.L902Q | 36 | 4 | preB |

**Table S2A.** AID/RAG expression signatures in correlation with the primary aberrations.

| **Primary aberration** | ***Total*** | ***AID^low^ RAG^low^*** | ***AID^high^ RAG^low^*** | ***AID^low^ RAG^high^*** | ***AID^high^ RAG^high^*** | ***P*** [a] |
|---|---|---|---|---|---|---|
| Total B-ALL | 156 | 29 (19%) | 51 (33%) | 49 (31%) | 27 (17%) | |
| NEG | 57 (37%) | 11 (19%) | 24 (42%) | 11 (19%) | 11 (19%) | |
| *BCR::ABL*$^{pos}$ | 53 (34%) | 9 (17%) | 11 (21%) | 21 (40%) | 12 (23%) | vs NEG: **0.0051** |
| *BCR::ABL*-like | 14 (9%) | 3 (21%) | 5 (36%) | 4 (29%) | 2 (14%) | vs NEG: 0.4625 |
| *MLL::AF4* | 5 (3%) | 2 (40%) | 0 (0%) | 3 (60%) | 0 (0%) | vs NEG: **0.0181** |
| *E2A::PBX* | 4 (3%) | 0 (0%) | 1 (25%) | 3 (75%) | 0 (0%) | vs NEG: 0.0853 |
| Complex karyotype | 15 (10%) | 1 (7%) | 7 (47%) | 6 (40%) | 1 (7%) | vs NEG: 0.3432 |
| Hyper-/Hypodiploid | 8 (5%) | 3 (38%) | 3 (38%) | 1 (13%) | 1 (13%) | vs NEG: 0.7919 |

**Table S2B.** AID/RAG expression signatures in correlation with the distribution of CNA profiles.

| **Subgroup** | ***Total*** | ***AID^low^ RAG^low^*** | ***AID^high^ RAG^low^*** | ***AID^low^ RAG^high^*** | ***AID^high^ RAG^high^*** | ***P*** [a] |
|---|---|---|---|---|---|---|
| | | | **TOTAL B-ALL** | | | |
| Total | 86 | 18 (21%) | 22 (26%) | 33 (38%) | 13 (15%) | |
| CNA$^{neg}$ | 27 (31%) | 6 (22%) | 12 (44%) | 9 (33%) | 0 (0%) | |
| 1 CNA | 22 (26%) | 3 (14%) | 6 (27%) | 8 (36%) | 5 (23%) | |

| | | | | | | |
|---|---|---|---|---|---|---|
| 2 CNAs | 20 (23%) | 3 (15%) | 4 (20%) | 9 (45%) | 4 (20%) | |
| ≥3 CNAs | 17 (20%) | 6 (35%) | 0 (0%) | 7 (41%) | 4 (24%) | |
| CNA$^{pos}$ | 59 (69%) | 12 (20%) | 10 (17%) | 24 (41%) | 13 (22%) | vs CNA$^{neg}$: **0.0414** |
| CNA$^{low}$ | 49 (57%) | 9 (18%) | 18 (37%) | 17 (35%) | 5 (10%) | |
| CNA$^{high}$ | 37 (43%) | 9 (24%) | 4 (11%) | 16 (43%) | 8 (22%) | vs CNA$^{low}$: **0.0221** |
| Age ≤ 40 y/o | 51 (59%) | 12 (24%) | 10 (20%) | 21 (41%) | 8 (16%) | |
| Age > 40 y/o | 35 (41%) | 6 (17%) | 12 (34%) | 12 (34%) | 5 (14%) | vs Age ≤ 40: 0.1829 |
| Age ≤ 40 y/o CNA$^{neg}$ | 20 (23%) | 6 (30%) | 6 (30%) | 8 (40%) | 0 (0%) | vs Age > 40 / CNA$^{neg}$: 0.0614 |
| Age ≤ 40 y/o CNA$^{pos}$ | 31 (36%) | 6 (19%) | 4 (13%) | 13 (42%) | 8 (26%) | vs Age > 40 / CNA$^{pos}$: 0.4516 |
| Age > 40 y/o CNA$^{neg}$ | 7 (8%) | 0 (0%) | 6 (86%) | 1 (14%) | 0 (0%) | |
| Age > 40 y/o CNA$^{pos}$ | 28 (33%) | 6 (21%) | 6 (21%) | 11 (39%) | 5 (18%) | vs Age > 40 / CNA$^{neg}$: **0.0247** |
| CNA$^{pos}$ *IKZF*$^{neg}$ | 24 (28%) | 3 (13%) | 9 (38%) | 6 (25%) | 6 (25%) | vs CNA$^{neg}$: 0.8639 |
| CNA$^{pos}$ *IKZF*$^{pos}$ | 35 (41%) | 9 (26%) | 1 (3%) | 18 (51%) | 7 (20%) | vs CNA$^{neg}$: **0.0005**<br>vs CNA$^{pos}$ / *IKZF*$^{neg}$: **0.0005** |
| CNA$^{high}$ *IKZF*$^{pos}$ | 26 (30%) | 7 (27%) | 0 (0%) | 13 (50%) | 6 (23%) | |
| CNA$^{high}$ *CDKN2A/B*$^{pos}$ *IKZF*$^{pos}$ | 14 (16%) | 1 (7%) | 0 (0%) | 8 (57%) | 5 (36%) | |
| CNA$^{high}$ *CDKN2A/B*$^{pos}$ *IKZF*$^{neg}$ | 16 (19%) | 2 (13%) | 5 (31%) | 4 (25%) | 5 (31%) | vs CNA$^{high}$ / *CDKN2A/B*$^{pos}$ / *IKZF*$^{pos}$: **0.0121** |
| | | | **NEG B-ALL** | | | |
| Total | 31 | 6 (19%) | 14 (45%) | 5 (16%) | 6 (19%) | |
| CNA$^{neg}$ | 10 (32%) | 2 (20%) | 7 (70%) | 1 (10%) | 0 (0%) | vs CNA$^{pos}$: 0.2435, **0.0317**[b] |
| CNA$^{pos}$ *IKZF*$^{neg}$ | 9 (29%) | 0 (0%) | 6 (67%) | 0 (0%) | 3 (33%) | vs CNA$^{neg}$: 0.3688, 0.0901[b] |
| CNA$^{pos}$ *IKZF*$^{pos}$ | 12 (39%) | 4 (33%) | 1 (8%) | 4 (33%) | 3 (25%) | vs CNA$^{neg}$: **0.0149, 0.0072**[b] |
| | | | ***BCR::ABL*$^{pos}$ B-ALL** | | | |
| Total | 28 | 5 (18%) | 2 (7%) | 17 (61%) | 4 (14%) | |

| | | | | | | |
|---|---|---|---|---|---|---|
| CNA$^{neg}$ | 6 (21%) | 1 (17%) | 2 (33%) | 3 (50%) | 0 (0%) | vs CNA$^{pos}$: **0.0124** |
| CNA$^{pos}$<br>*IKZF*$^{neg}$ | 4 (14%) | 1 (25%) | 0 (0%) | 1 (25%) | 2 (50%) | vs CNA$^{neg}$: 0.4386 |
| CNA$^{pos}$<br>*IKZF*$^{pos}$ | 18 (64%) | 3 (17%) | 0 (0%) | 13 (72%) | 2 (11%) | vs CNA$^{neg}$: **0.0156** |

[a] computed by chi-squared test. The comparison involves *AID*$^{high}$ / *RAG*$^{low}$ and *AID*$^{low}$ / *RAG*$^{high}$ signatures.
[b] computed by chi-squared test. The comparison involves *AID*$^{high}$ / *RAG*$^{low}$ and *AID*$^{high}$ / *RAG*$^{high}$ signatures.

Abbreviations: *RAG*, Recombination Activating Gene; *AID,* Activation Induced Cytidine Deaminase; CNA, copy number alteration

**Table S3A.** Frequency of CNA mutation burden and specific gene deletions in genetic subgroups of the studied cohort.

| | **Total B-ALL**<br>n=94 | **NEG B-ALL**<br>n=35 | ***BCR::ABL1*$^{pos}$**<br>n=30 | ***BCR::ABL1-like***<br>n=12 |
|---|---|---|---|---|
| **CNA$^{neg}$** | 28<br>30% | 11<br>39% of CNA$^{neg}$<br>31% of NEG | 6<br>21% of CNA$^{neg}$<br>20% of *BCR::ABL* | 2<br>7% of CNA$^{neg}$<br>17% of *BCR::ABL*-like |
| **1 CNA** | 25<br>27% | 9<br>36% of 1 CNA<br>26% of NEG | 8<br>32% of 1 CNA<br>27% of *BCR::ABL* | 3<br>12% of 1 CNA<br>25% of *BCR::ABL*-like |
| **2 CNAs** | 22<br>23% | 10<br>45% of 2 CNAs<br>29% of NEG | 7<br>32% of 2 CNAs<br>23% of *BCR::ABL* | 4<br>18% of 2 CNAs<br>33% of *BCR::ABL*-like |
| **≥3 CNAs** | 19<br>20% | 5<br>26% of ≥3 CNAs<br>14% of NEG | 9<br>47% of ≥3 CNAs<br>30% of *BCR::ABL* | 3<br>16% of ≥3 CNAs<br>25% of *BCR::ABL*-like |
| ***IKZF1*** | 41$^{\#}$<br>44% | 14<br>29% of *IKZF1*<br>40% of NEG | 27<br>55% of *IKZF1*<br>67% of *BCR::ABL* | 6<br>12% of *IKZF1*<br>50% of *BCR::ABL*-like |
| ***CDKN2A/B*** | 32<br>34% | 11<br>34% of *CDKN2A/B*<br>31% of NEG | 12<br>38% of *CDKN2A/B*<br>40% of *BCR::ABL* | 4<br>13% of *CDKN2A/B*<br>33% of *BCR::ABL*-like |
| ***PAX5*** | 15<br>16% | 4<br>27% of *PAX5*<br>11% of NEG | 8<br>53% of *PAX5*<br>27% of *BCR::ABL* | 2<br>13% of *PAX5*<br>17% of *BCR::ABL*-like |
| ***EBF1*** | 14<br>15% | 7<br>50% of *EBF1*<br>20% of NEG | 3<br>21% of *EBF1*<br>10% of *BCR::ABL* | 3<br>21% of *EBF1*<br>25% of *BCR::ABL*-like |
| ***ETV6*** | 14<br>15% | 5<br>36% of *ETV6*<br>14% of NEG | 2<br>14% of *ETV6*<br>7% of *BCR::ABL* | 3<br>21% of *ETV6*<br>25% of *BCR::ABL*-like |
| ***BTG1*** | 7<br>7% | 2<br>29% of *BTG1*<br>6% of NEG | 3<br>43% of *BTG1*<br>10% of *BCR::ABL* | 2<br>29% of of *BTG1*<br>17% of *BCR::ABL*-like |
| ***RB1*** | 5<br>5% | 2<br>40% of *RB1*<br>6% of NEG | 2<br>40% of *RB1*<br>7% of *BCR::ABL* | 0<br>0% of *RB1*<br>0% of *BCR::ABL*-like |

**Table S3B**. Correlation between CNA mutation burden and deletions in each gene.

| | **Total CNA**$^{pos}$ **ALL** n=66 | **1 CNA** n=25 | **2 CNAs** n=22 | **≥3 CNAs** n=19 |
|---|---|---|---|---|
| ***IKZF1*** | 41# 62% | 12 29% of *IKZF* 48% of 1 CNA | 11 27% of *IKZF* 50% of 2 CNAs | 18 44% of *IKZF* 95% of ≥3 CNAs |
| ***CDKN2A/B*** | 32 48% | 7 22% of *CDKN2A/B* 28% of 1 CNA | 13 41% of *CDKN2A/B* 59% of 2 CNAs | 12 38% of *CDKN2A/B* 63% of ≥3 CNAs |
| ***PAX5*** | 15 23% | 1 7% of *PAX5* 4% of 1 CNA | 3 20% of *PAX5* 14% of 2 CNAs | 11 73% of *PAX5* 58% of ≥3 CNAs |
| ***EBF1*** | 14 21% | 2 14% of *EBF1* 8% of 1 CNA | 7 50% of *EBF1* 32% of 2 CNAs | 5 36% of *EBF1* 26% of ≥3 CNAs |
| ***ETV6*** | 14 21% | 3 21% of *ETV6* 12% of 1 CNA | 7 50% of *ETV6* 32% of 2 CNAs | 4 29% of *ETV6* 21% of ≥3 CNAs |
| ***BTG1*** | 7 11% | 0 0% of *BTG1* 0% of 1 CNA | 2 29% of *BTG1* 9% of 2 CNAs | 5 71% of *BTG1* 26% of ≥3 CNAs |
| ***RB1*** | 5 8% | 0 0% of *RB1* 0% of 1 CNA | 0 0% of *RB1* 0% of 2 CNAs | 5 100% of *RB1* 26% of ≥3 CNAs |

#8 *IKZF1*-mutated patients were detected with the RT-PCR assay and their number of CNAs was not evaluated. These cases are not included in both tables.

**Table S4.** Univariate analysis of patients' outcome in subgroups according to CNA status.

| **End point and variables** | ***Total*** | A.<br>**CNA$^{neg}$** | B.<br>**1 CNA** | A. vs B.<br>***P*** | C.<br>**CNA$^{high}$** | B. vs C.<br>***P*** | D.<br>**1 CNA / *IKZF*$^{pos}$** | E.<br>**CNA$^{high}$ / *IKZF*$^{pos}$** | D. vs E.<br>***P*** | F.<br>**CNA$^{high}$ / *IKZF*$^{neg}$** | E. vs F.<br>***P*** | G.<br>**CNA$^{pos}$ other than CNA$^{high}$ / *IKZF*$^{pos}$** | E. vs G.<br>***P*** |
|---|---|---|---|---|---|---|---|---|---|---|---|---|---|
| **TOTAL B-ALL** | | | | | | | | | | | | | |
| **CR**<br>No. of patients<br>(%) | 73/88<br>83% | 22/26<br>85% | 23/24<br>96% | 0.2004[†] | 28/38<br>74% | **0.0247**[†] | 12/12<br>100% | 20/28<br>71% | **0.0404**[†] | 8/12<br>67% | 0.5214[†] | 31/34<br>91% | **0.0451**[†] |
| **OS**<br>No. of patients<br>4-year rate ± SE | 92<br>36 ± 6 % | 27<br>43 ± 10 % | 25<br>45 ± 11 % | 0.5125[#] | 40<br>20 ± 7 % | **0.0139**[#] | 11<br>56 ± 17 % | 28<br>9 ± 6 % | **0.0010**[#] | 12<br>42 ± 14 % | **0.0327**[#] | 37<br>43 ± 9 % | **0.0035**[#] |
| **RFS**<br>No. of patients<br>4-year rate ± SE | 73<br>41 ± 7 % | 22<br>43 ± 10 % | 22<br>48 ± 12 % | 0.5822[#] | 27<br>20 ± 10 % | 0.1167[#] | 12<br>52 ± 16 % | 19<br>14 ± 9 % | **0.0395**[#] | 8<br>39 ± 20 % | **0.0026**[#] | 30<br>45 ± 10 % | **0.0061**[#] |
| **NEG B-ALL** | | | | | | | | | | | | | |
| **CR**<br>No. of patients<br>(%) | 28/32<br>88% | 9/10<br>90% | 9/9<br>100% | 0.5263[†] | 10/13<br>77% | 0.1857[†] | 4/4<br>100% | 7/9<br>78% | 0.3054[†] | 3/5<br>60% | 0.4805[†] | 12/13<br>92% | 0.3584[†] |
| **OS**<br>No. of patients<br>4-year rate ± SE | 34<br>28 ± 9 % | 10<br>53 ± 17 % | 9<br>49 ± 19 % | 0.5796[#] | 15<br>7 ± 6 % | **0.0028**[#] | 3<br>33 ± 27 % | 10<br>0 % | 0.0667[#] | 5<br>20 ± 18 % | 0.0797[#] | 14<br>28 ± 13 % | **0.0089**[#] |
| **RFS**<br>No. of patients<br>4-year rate ± SE | 28<br>25 ± 10 % | 9<br>42 ± 20 % | 9<br>44 ± 19 % | 0.8310[#] | 10<br>0 % | **0.0228**[#] | 4<br>38 ± 29 % | 7<br>0 % | 0.1321[#] | 3<br>0 % | **0.0072**[#] | 12<br>31 ± 17 % | **0.0048**[#] |
| ***BCR::ABL1*$^{pos}$ B-ALL** | | | | | | | | | | | | | |
| **CR**<br>No. of patients<br>(%) | 24/30<br>80% | 4/6<br>67% | 8/8<br>100% | 0.1648[†] | 12/16<br>75% | 0.1713[†] | 6/6<br>100% | 10/14<br>71% | 0.1432[†] | 2/2<br>100% | 0.3827[†] | 10/10<br>100% | 0.0942[†] |
| **OS**<br>No. of patients<br>4-year rate ± SE | 29<br>47 ± 10 % | 6<br>33 ± 19 % | 8<br>86 ± 13 % | **0.0410**[#] | 15<br>33 ± 13 % | **0.0283**[#] | 6<br>80 ± 18 % | 13<br>20 ± 12 % | **0.0270**[#] | 2<br>100 % | **0.0355**[#] | 10<br>89 ± 10 % | **0.0032**[#] |

| | | | | | | | | | | | | | |
|---|---|---|---|---|---|---|---|---|---|---|---|---|---|
| **RFS** | | | | | | | | | | | | | |
| No. of patients | 23 | 4 | 8 | 0.6115[#] | 11 | 0.0961[#] | 6 | 9 | 0.0884[#] | 2 | 0.0739[#] | 10 | **0.0167**[#] |
| 4-year rate ± SE | 65 ± 11 % | 75 ± 22 % | 86 ± 13 % | | 44 ± 17 % | | 80 ± 18 % | 29 ± 17 % | | 100 % | | 89 ± 10 % | |

[†] computed by chi-squared or Fisher's exact test

[#] computed by log-rank test

Abbreviations: CR, complete remission; OS, overall survival; RFS, relapse-free survival; SE, standard error; CNA, copy number alteration;

**Table S5A.** Frequency of patients with relapse and those remaining in remission – in subgroups according to mutator enzyme expression.

| Outcome | *AID* expression | | | | *RAG* expression | | | |
|---|---|---|---|---|---|---|---|---|
| | *Total* | *AID*$^{low}$ | *AID*$^{high}$ | *P* | *Total* | *RAG*$^{low}$ | *RAG*$^{high}$ | *P* |
| CNA$^{neg}$ | | | | | | | | |
| CR | 15 | 12 (80%) | 3 (20%) | **0.0195**[a] | 14 | 6 (43%) | 8 (57%) | **0.0110**[d] |
| REL | 7 | 2 (29%) | 5 (71%) | | 7 | 7 (100%) | 0 (0%) | |
| CNA$^{pos}$ | | | | | | | | |
| CR | 19 | 12 (63%) | 7 (37%) | **0.0082**[b] | 21 | 7 (33%) | 14 (67%) | **0.0133**[e] |
| REL | 25 | 16 (64%) | 9 (36%) | 0.0949[c] | 26 | 13 (50%) | 13 (50%) | **0.0163**[f] |

[a] *AID*$^{low}$ vs *AID*$^{high}$ and CR vs REL, in CNA$^{neg}$ subgroup
[b] CNA$^{neg}$ vs CNA$^{pos}$ and CR vs REL, in *AID*$^{low}$ subgroup
[c] *AID*$^{low}$ vs *AID*$^{high}$ and CNA$^{neg}$ vs CNA$^{pos}$, in REL subgroup
[d] *RAG*$^{low}$ vs *RAG*$^{high}$ and CR vs REL, in CNA$^{neg}$ subgroup
[e] CNA$^{neg}$ vs CNA$^{pos}$ and CR vs REL, in *RAG*$^{high}$ subgroup
[f] *RAG*$^{low}$ vs *RAG*$^{high}$ and CNA$^{neg}$ vs CNA$^{pos}$, in REL subgroup
All p-values were calculated with chi-squared test

Abbreviations: *RAG*, Recombination Activating Gene; *AID*, Activation Induced Cytidine Deaminase; CNA, copy number alteration; CR, complete remission; REL, relapse

**Table S5B.** Univariate analysis of CNA$^{neg}$ patients' outcomes according to mutator enzyme expression.

| End points and variables | *Total* | *AID* expression | | | *RAG* expression | | |
|---|---|---|---|---|---|---|---|
| | | *AID*$^{low}$ | *AID*$^{high}$ | *P* | *RAG*$^{low}$ | *RAG*$^{high}$ | *P* |
| **CR** | | | | | | | |
| No. of patients | 21/25 | 14/15 | 8/11 | 0.1876† | 13/16 | 8/9 | 0.5423† |
| (%) | 84% | 93% | 72% | | 81% | 89% | |
| **OS** | | | | | | | |
| No. of patients | 25 | 14 | 11 | **0.0306**# | 15 | 9 | **0.0441**# |
| 4-year rate ± SE | 52 ± 11 % | 67 ± 14 % | 30 ± 15 % | | 34 ± 13 % | 76 ± 15 % | |
| **RFS** | | | | | | | |
| No. of patients | 22 | 14 | 8 | **0.0158**# | 13 | 8 | **0.0105**# |
| 4-year rate ± SE | 59 ± 14 % | 84 ± 10 % | 21 ± 18 % | | 32 ± 17 % | 100 % | |

† computed by chi-squared or Fisher's exact test
# computed by log-rank test

Abbreviations: CR, complete remission; OS, overall survival; RFS, relapse-free survival; SE, standard error; CNA, copy number alteration

**Table S5C.** Multivariate analysis of CNA$^{neg}$ patients' outcomes according to mutator enzyme expression, adjusted for age and WBC.

| **End points and variables** | ***AID*$^{high}$** | ***RAG*$^{high}$** |
|---|---|---|
| **CR** | | |
| OR (95% CI) | 0.73 (0.39-1.36) | 0.87 (0.50-1.53) |
| *P* * | 0.3038 | 0.6272 |
| **OS** | | |
| HR (95% CI) | 1.27 (0.96-1.68) | 1.13 (0.90-1.43) |
| *P* ‡ | 0.0886 | 0.3034 |
| **RFS** | | |
| HR (95% CI) | 1.58 (1,13-2,22) | 0.79 (0.63-0.98) |
| *P* ‡ | **0.0080** | **0.0329** |

* computed from General Linear Model

‡ computed by Cox proportional hazard regression

Abbreviations: WBC, white blood count; CR, complete remission; OS, overall survival; RFS, relapse-free survival; CNA, copy number alteration; HR, hazard ratio; OR, odds ratio; 95% CI, 95% confidence interval

## Supplementary figures

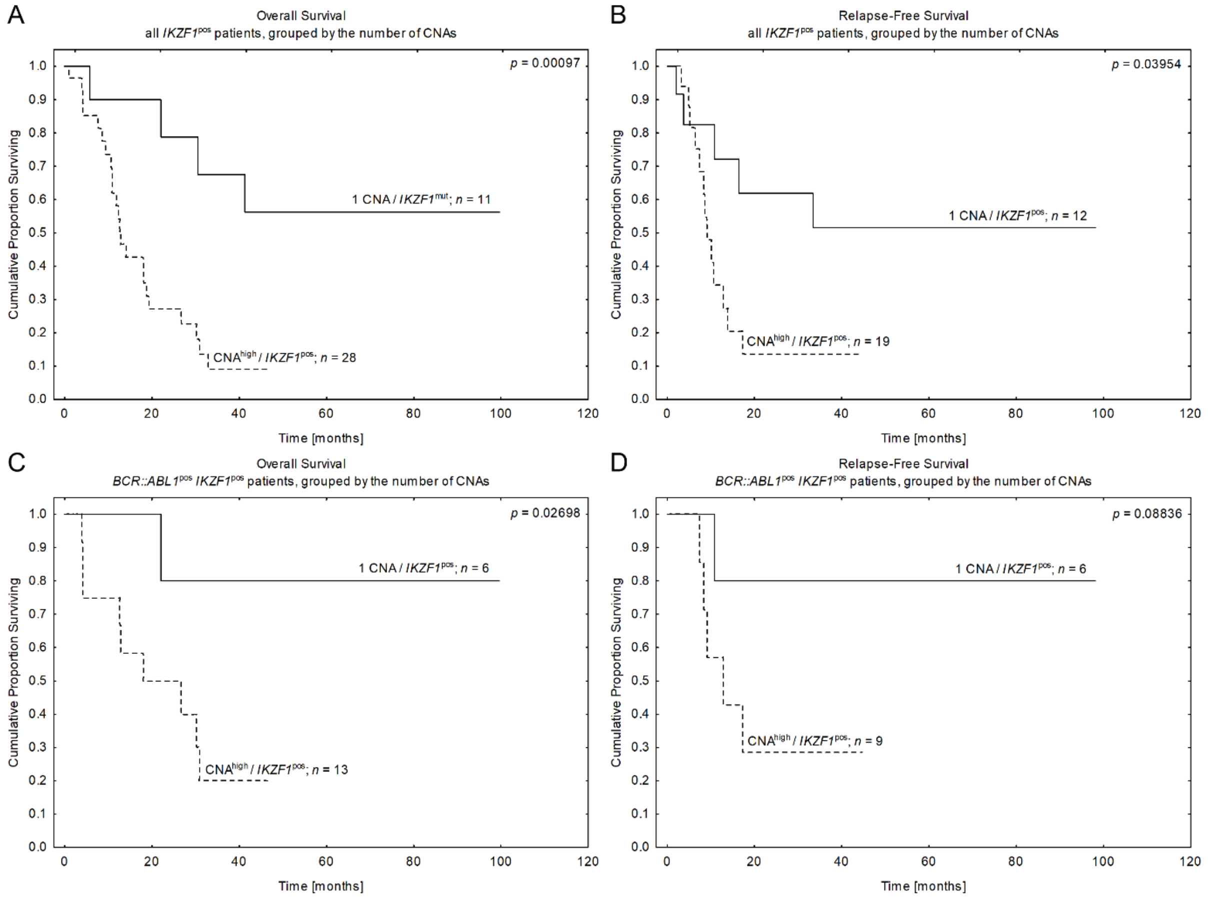


**Figure S1**. Outcome of *IKZF1*-positive patients according to the number of CNAs. Kaplan-Meier estimates for the probability of overall survival and relapse-free survival in *IKZF1*-positive patients according to the number of copy number alterations (CNAs). CNA$^{high}$ – ≥2 detected CNAs. (A,B)—overall survival and relapse-free survival in all *IKZF1*-mutated patients. (C,D)—overall survival and

relapse-free survival in *BCR::ABL1*-positive *IKZF1*-positive patients only. *n*—number of patients, *p*—p-value.

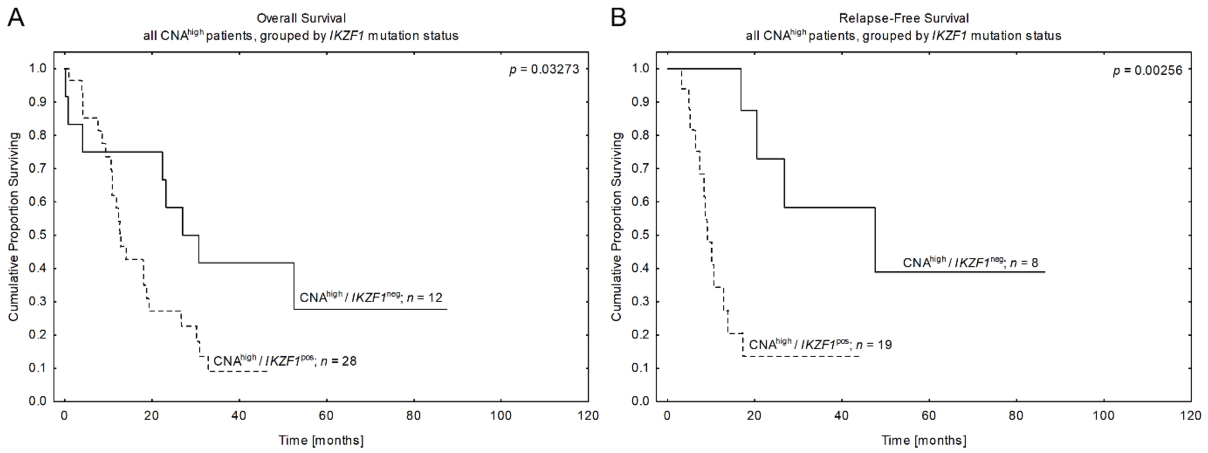


**Figure S2**. Outcome of CNA$^{high}$ patients according to *IKZF1* mutation status. Kaplan-Meier estimates for the probability of (A) overall survival and (B) relapse-free survival in CNA$^{high}$ patients according to *IKZF1* mutation status. CNA$^{high}$ – ≥2 detected CNAs. *n*—number of patients, *p*—*p*-value.

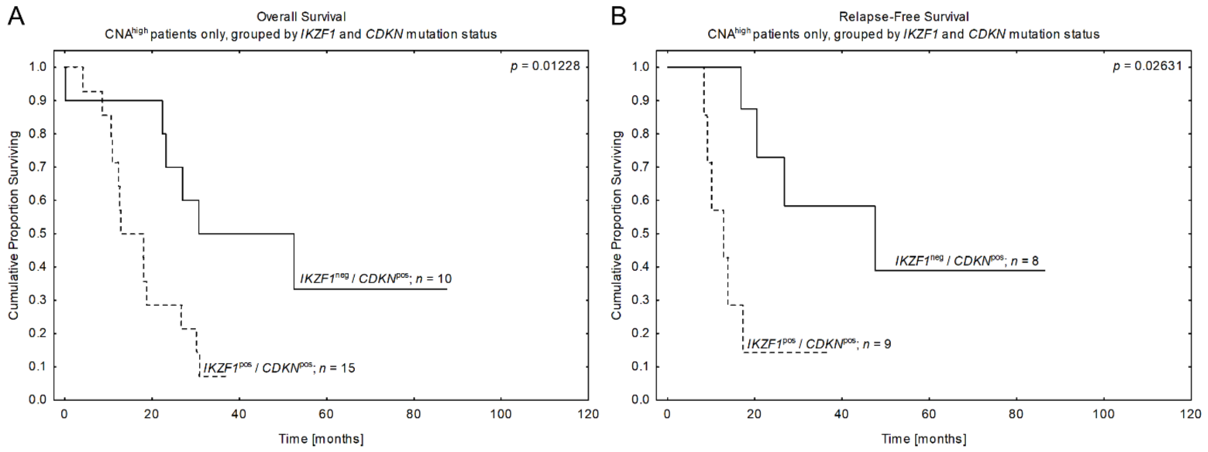


**Figure S3**. Outcome of CNA$^{high}$ patients according to *IKZF1* and *CDKN2A/B* mutation status. Kaplan-Meier estimates for the probability of (A) overall survival and (B) relapse-free survival in CNA$^{high}$ *CDKN2A/B*$^{pos}$ patients according to *IKZF1* mutation status. The graph only contains *CDKN2A/B*$^{pos}$ patients. *n*—number of patients, *p*—*p*-value.

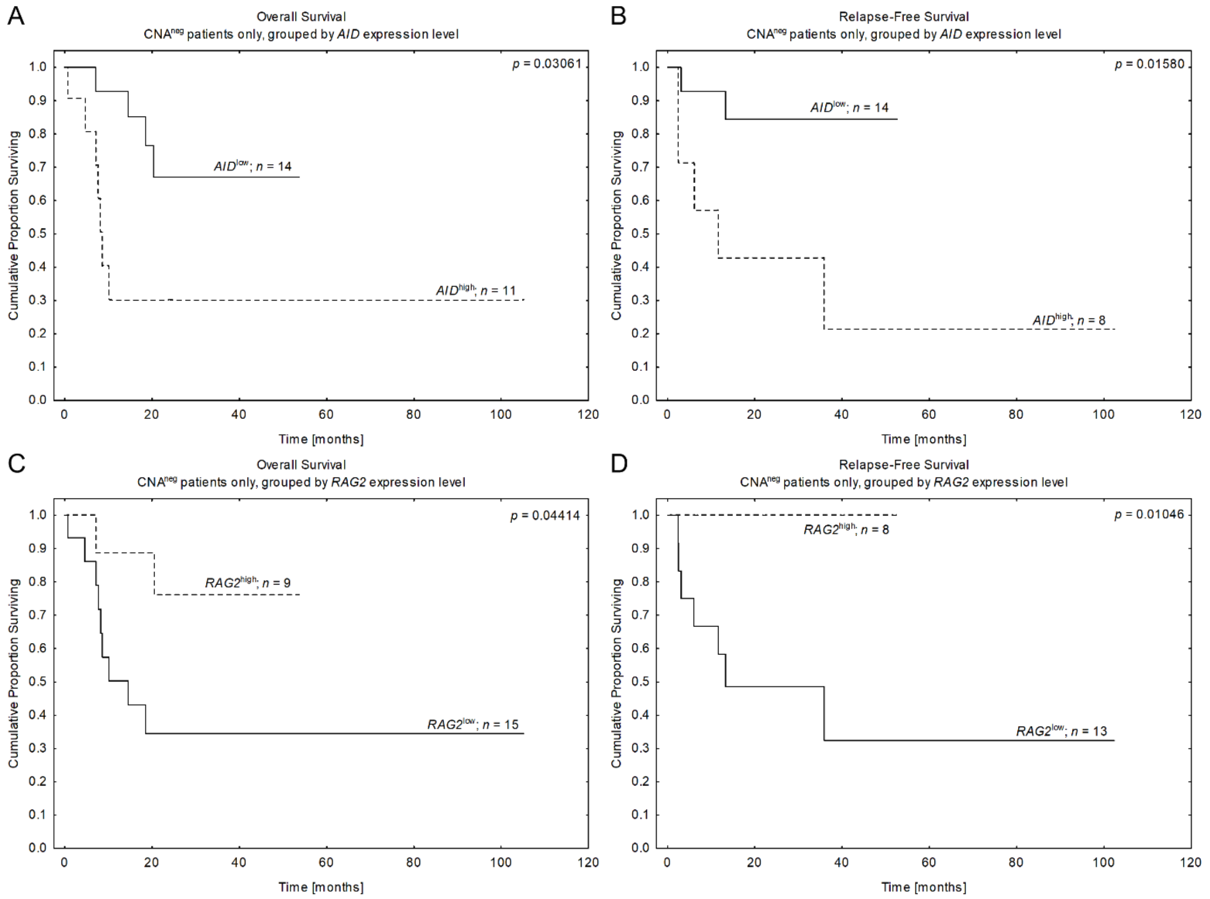


**Figure S4**. Outcome of CNA$^{neg}$ patients according to *RAG2* and *AID* expression. Kaplan-Meier estimates for the probability of overall survival and relapse-free survival in CNA$^{neg}$ patients according to the *AID* and *RAG2* expression levels. (A), (B) – overall survival and relapse-free survival in CNA$^{neg}$ patients according to the *AID* expression level. (C), (D) – overall survival and relapse-free survival in CNA$^{neg}$ patients according to the *RAG2* expression level. CNA$^{neg}$ – 0 detected copy number alterations, n – number of patients, p – p-value

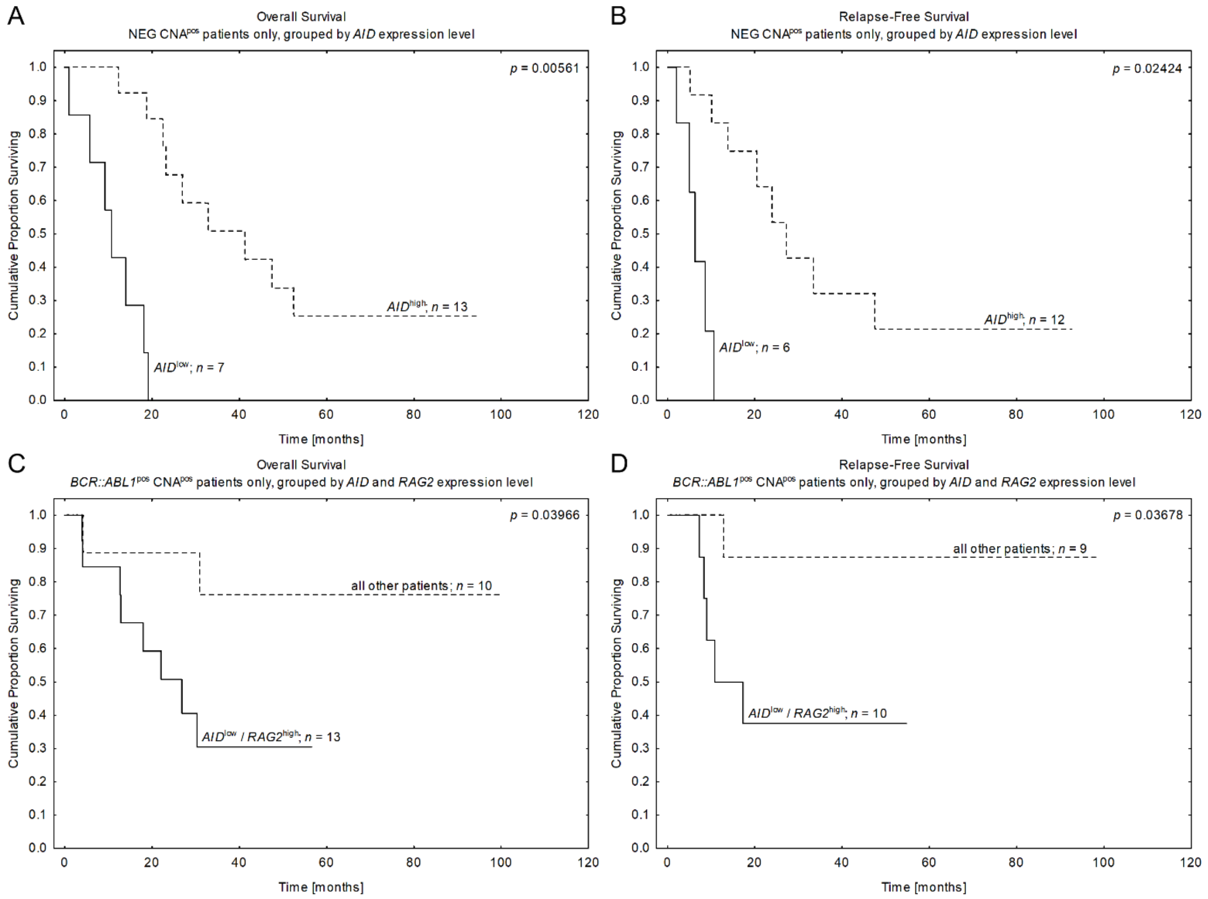


**Figure S5**. Outcome of CNA$^{pos}$ patients according to *RAG2* and *AID* expression. Kaplan-Meier estimates for the probability of overall survival and relapse-free survival in CNA$^{pos}$ patients according to the *AID* and *RAG2* expression levels. (A), (B) – overall survival and relapse-free survival in NEG CNA$^{pos}$ patients according to the *AID* expression level. (C), (D) – overall survival and relapse-free survival in *BCR::ABL1*$^{pos}$ CNA$^{pos}$ patients according to the *RAG2* and *AID* expression levels. CNA$^{pos}$ – at least 1 detected copy number alteration, n – number of patients, p – p-value

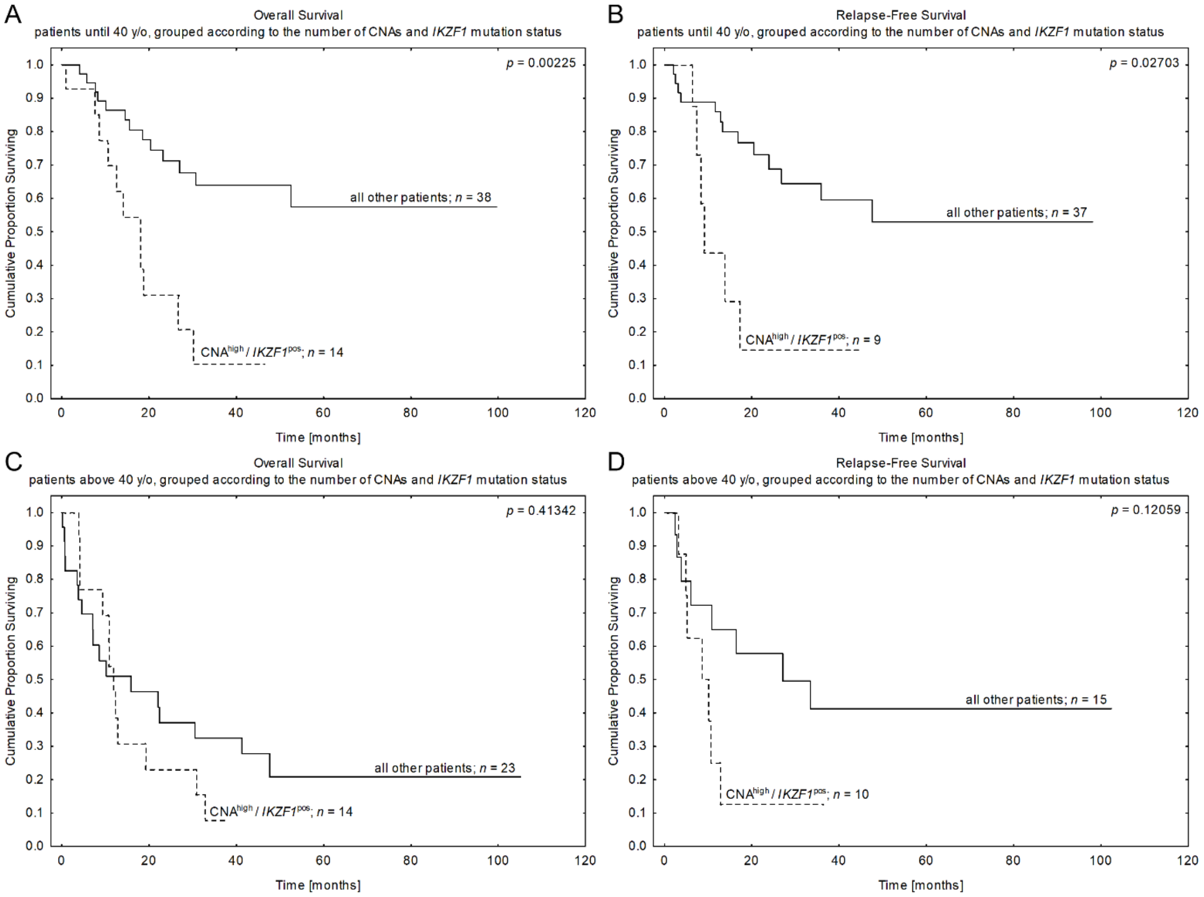


**Figure S6**. Outcome of younger and older patients according to CNA profile. Kaplan-Meier estimates for the probability of overall survival and relapse-free survival in all studied patients according to the CNA profile, divided into younger (≤ 40y.) and older (>40y.) patients. (A), (B) – overall survival and relapse-free survival in younger patients, grouped into CNA$^{high}$ / *IKZF1*$^{pos}$ and all other CNA profiles. (C), (D) – overall survival and relapse-free survival in older patients, grouped into CNA$^{high}$ / *IKZF1*$^{pos}$ and all other CNA profiles. CNA$^{high}$ – at least 2 detected copy number alteration, n – number of patients, p – p-value

## References


1. Shochat, C.; Tal, N.; Bandapalli, O.R.; Palmi, C.; Ganmore, I.; te Kronnie, G.; Cario, G.; Cazzaniga, G.; Kulozik, A.E.; Stanulla, M.; et al. Gain-of-Function Mutations in Interleukin-7 Receptor-α (IL7R) in Childhood Acute Lymphoblastic Leukemias. *Journal of Experimental Medicine* **2011**, *208*, 901–908, doi:10.1084/jem.20110580.

2. Boer, J.M.; Koenders, J.E.; Van Der Holt, B.; Exalto, C.; Sanders, M.A.; Cornelissen, J.J.; Valk, P.J.M.; Den Boer, M.L.; Rijneveld, A.W. Expression Profiling of Adult Acute Lymphoblastic Leukemia Identifies a BCR-ABL1-like Subgroup Characterized by High Non-Response and Relapse Rates. *Haematologica* **2015**, *100*, 261–264, doi:10.3324/haematol.2014.117424.

3. Holmfeldt, L.; Wei, L.; Diaz-Flores, E.; Walsh, M.; Zhang, J.; Ding, L.; Payne-Turner, D.; Churchman, M.; Andersson, A.; Chen, S.C.; et al. The Genomic Landscape of Hypodiploid Acute Lymphoblastic Leukemia. *Nat Genet* **2013**, *45*, 242–252, doi:10.1038/ng.2532.

4. Iacobucci, I.; Iraci, N.; Messina, M.; Lonetti, A.; Chiaretti, S.; Valli, E.; Ferrari, A.; Papayannidis, C.; Paoloni, F.; Vitale, A.; et al. IKAROS Deletions Dictate a Unique Gene Expression Signature

in Patients with Adult B-Cell Acute Lymphoblastic Leukemia. *PLoS One* **2012**, *7*, doi:10.1371/journal.pone.0040934.

5. Hertzberg, L.; Vendramini, E.; Ganmore, I.; Cazzaniga, G.; Schmitz, M.; Chalker, J.; Shiloh, R.; Iacobucci, I.; Shochat, C.; Zeligson, S.; et al. Down Syndrome Acute Lymphoblastic Leukemia, a Highly Heterogeneous Disease in Which Aberrant Expression of CRLF2 Is Associated with Mutated JAK2: A Report from the International BFM Study Group. *Blood* **2010**, *115*, 1006–1017, doi:10.1182/blood-2009-08.

6. Rao, X.; Huang, X.; Zhou, Z.; Lin, X. An Improvement of the 2ˆ(-Delta Delta CT) Method for Quantitative Real-Time Polymerase Chain Reaction Data Analysis. *Biostat Bioinforma Biomath* **2013**, *3*, 71–85.

7. Kwon, M.; Martínez-Laperche, C.; Infante, M.; Carretero, F.; Balsalobre, P.; Serrano, D.; Gayoso, J.; Pérez-Corral, A.; Anguita, J.; Díez-Martín, J.L.; et al. Evaluation of Minimal Residual Disease by Real-Time Quantitative PCR of Wilms' Tumor 1 Expression in Patients with Acute Myelogenous Leukemia after Allogeneic Stem Cell Transplantation: Correlation with Flow Cytometry and Chimerism. *Biology of Blood and Marrow Transplantation* **2012**, *18*, 1235–1242, doi:10.1016/j.bbmt.2012.01.012.

8. Iacobucci, I.; Lonetti, A.; Messa, F.; Cilloni, D.; Arruga, F.; Ottaviani, E.; Paolini, S.; Papayannidis, C.; Piccaluga, P.P.; Giannoulia, P.; et al. Expression of Spliced Oncogenic Ikaros Isoforms in Philadelphia-Positive Acute Lymphoblastic Leukemia Patients Treated with Tyrosine Kinase Inhibitors: Implications for a New Mechanism of Resistance. *Blood* **2008**, *112*, 3847–3855, doi:10.1182/blood-2007-09-112631.
